# The preparation and properties of 1,1-difluorocyclopropane derivatives

**DOI:** 10.3762/bjoc.17.25

**Published:** 2021-01-26

**Authors:** Kymbat S Adekenova, Peter B Wyatt, Sergazy M Adekenov

**Affiliations:** 1 School of Biological and Chemical Sciences, Queen Mary University of London, Mile End Road, London, E1 4NS, UK; 2 JSC "International Research and Production Holding "Phytochemistry", Karaganda city, 100009, Republic of Kazakhstan

**Keywords:** biological activity, cyclopropanation, difluorocyclopropane derivatives, synthesis

## Abstract

Recently, the functionalization of organic molecules with fluorine substituents has grown rapidly due to its applications in such fields as medicine, agriculture or materials sciences. The aim of this article is to review the importance of 1,1-difluorocyclopropane derivatives in synthesis. It will examine the role of the fluorine substituents in both ring-forming and ring-opening reactions, as well as methods for obtaining difluorocyclopropanes as single enantiomers. Several examples are provided to highlight the biological importance of this class of compounds.

## Introduction

The chemistry of cyclopropane derivatives is one of the most intensively developing fields of organic chemistry. In the past decade there have been made many investigations to develop new chemo-, regio- and stereoselective methods for the synthesis and transformations of cyclopropane derivatives. These investigations gained a significant interest, because cyclopropane and cyclopropene fragments are present in the structures of many biologically active substances, such as antibiotics, anticancer, and antimycotic preparations, controllers of plant growth and fruit ripening, and insecticides. Geminal dihalocyclopropanes, especially the fluoro derivatives, form an important class of organic compounds, which have the ability to participate in synthetically useful reactions due to the presence of both, ring strain and of the *gem*-dihalomethylene fragment. Thus, they are of interest not only for the direct application as biologically active substances and functional materials but also as precursors to other fluorine-containing compounds [1–2]. Fluorine forms stable bonds to carbon and due to its high electronegativity it can profoundly modify the physicochemical properties of the parent molecules. In biologically active materials fluorine substituents can affect the charge distribution, electrostatic surface, and solubility of chemical entities, thus often leading to useful outcomes. Incorporating a fluorine group into natural compounds has been widely accepted as a powerful tool for discovering new drugs and agrochemicals. The number of medicinal preparations containing at least one fluorine atom in the structure is now very high [3–5].

In this review we give an overview of the chemistry of 1,1-difluorocyclopropanes. First, we discuss the synthetic routes to *gem-*fluorocyclopropane derivatives. Then, we review the chemical transformations, emphasizing ring-opening reactions. Finally, we survey the biological activity of significant molecules that possess the 1,1-difluorocyclopropane fragment in the structure. A number of previous reviews dealing with the synthesis and applications of difluorocyclopropanes are available [2,6–8]. Here we will focus on selected synthetically and biologically useful examples.

## Review

### 1 Synthesis of 1,1-difluorocyclopropanes

**Scheme 1:**
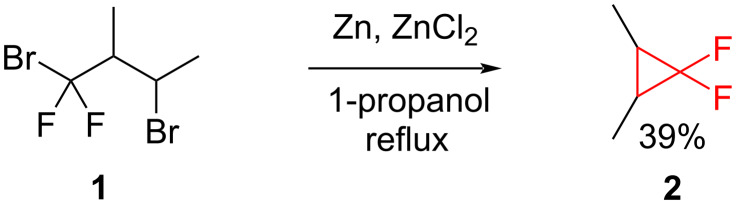
Synthesis of 1,1-difluoro-2,3-dimethylcyclopropane (**2**).

An early work on the synthesis and reactivity of fluorinated cyclopropanes was described by Atkinson in 1952 [9], followed by Tarrant [10], and Misani [11]. Tarrant, Lovelace and Lilyquist synthesized 1,1-difluoro-2,3-dimethylcyclopropane (**2**) by a reductive debromination using zinc metal (Scheme 1) [10].

After 1960 further methods of generating difluorocarbenes became available. These methods contributed to the synthesis of a wide variety of fluorinated cyclopropanes. In 2003, two reviews by Dolbier [7] and Fedorynski [8] were published on the methods of synthesis and use of difluorocyclopropanes in organic synthesis. They discussed in detail the various approaches for the synthesis of difluorocyclopropanes, so in this review we will supplement this information by methods for the synthesis of difluorocyclopropanes, paying particular attention to the practical methods of synthesis and transformation.

Three main approaches to the preparation of difluorocyclopropane and its derivatives can be distinguished: carbene and non-carbene methods of cyclopropanation along with functional group transformations of existing cyclopropanes.

The most popular route to prepare fluorocyclopropanes is to generate fluorine-containing carbenes (or carbenoids), which then react with multiple bonds, resulting in cyclopropanation. One of the important properties of fluorine-containing carbenes and carbenoids is their electrophilicity, which is a result of the high electronegativity of fluorine. Also, fluorine has an +M effect which tends to reduce the reactivity of the carbenes. The carbene-based methods typically give the highest yields when alkenes with electron-donating substituents are used. There are few examples in which the cyclopropanation by carbene methods of electron-deficient alkenes containing substituents with a large −M effect (for example, CO_2_R, COR, CN, SO_2_R) were successful. Therefore, alternative methods such as intramolecular cyclizations, exchange fluorination, and transformation of functional groups in fluorinated rings have been developed in order to provide access to fluorinated cyclopropanes with electron-withdrawing substituents.

#### 1.1 Difluorocarbene methods with non-metal sources

**Scheme 2:**
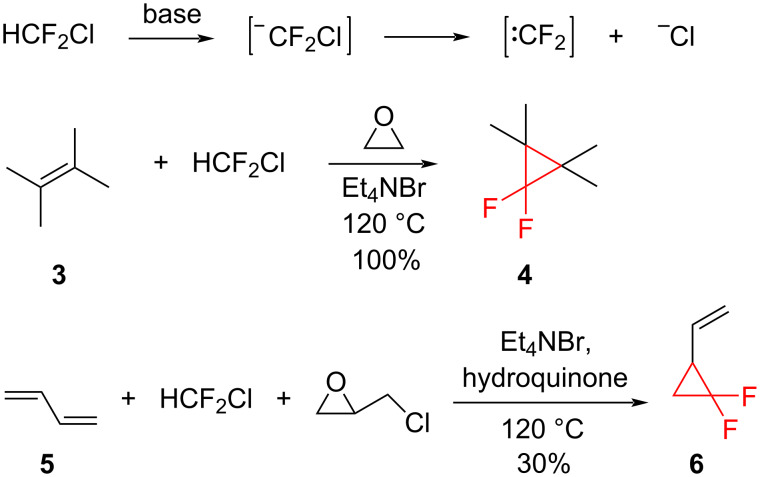
Cyclopropanation via dehydrohalogenation of chlorodifluoromethane.

Difluorocarbene chemistry was first reported by Doering in 1954 [12]. The lone electron pairs on the fluorine substituents interact with the carbene center, making the structure stabilized [13]. Difluorocyclopropanes **4** were synthesized from the reaction of halodifluoromethanes and alkenes (Scheme 2). The elimination of hydrogen halide from the halodifluoromethane under basic conditions (metal alkoxide or alkyllithium) generated difluorocarbene [14–15]. The low yields of the product have been attributed to the facile addition of the strong bases to difluorocarbene. The yields were best in the reactions with electron-rich alkenes and when a low concentration of the base was used to minimize the destruction of difluorocarbene. The use of oxirane or epichlorohydrin as hydrogen halide scavengers avoided the need for a stoichiometric amount of the strong base [16–17]. The opening of the oxirane ring by bromide ions under homogeneous conditions generated a bromoalkoxide ion which then acted as the base, leading to cyclopropanes **4** and **6** (Scheme 2). However, the harsh conditions needed (high temperatures, autoclave) limited the approach.

**Scheme 3:**
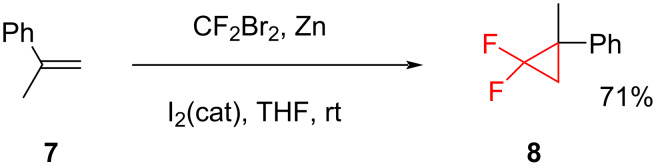
Difluorocyclopropanation of methylstyrene **7** using dibromodifluoromethane and zinc.

In the case of electron-rich alkenes dibromodifluoromethane is a suitable source of difluorocarbene. However, the same reagent produces low yields in the reactions with electron-deficient alkenes. Dolbier et al. reported the cyclopropanation of α-methylstyrene (**7**) using dibromodifluoromethane and zinc dust in the presence of iodine (Scheme 3) [18].

**Scheme 4:**
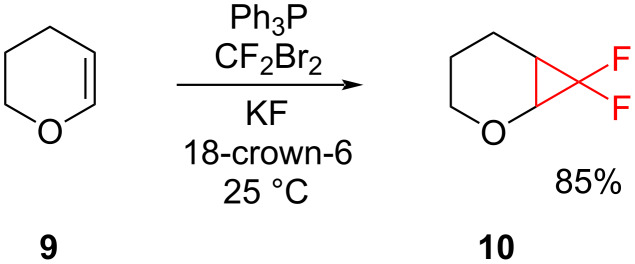
Synthesis of difluorocyclopropanes from the reaction of dibromodifluoromethane and triphenylphosphine.

The reduction of dibromodifluoromethane was also used for the approach of Burton and Naae (Scheme 4), which is again suitable for electron-rich alkenes [19]. Dibromodifluoromethane reacted with triphenylphosphine to give a phosphonium salt, which then decomposed to difluorocarbene. The yields from this method were increased when potassium fluoride and 18-crown-6 were added to the reaction mixture [20].

**Dehydrohalogenation of dichlorodifluoromethane under phase-transfer catalysis:** Difluorocarbene can be generated from chlorodifluoromethane by phase-transfer catalysis (PTC) through the reaction with NaOH or KOH, or a solid base, using a tetraalkylammonium salt as the catalyst. However, the resulting difluorocarbene is ineffective for the cyclopropanation of alkenes. This is because the intermediate chlorodifluoromethyl anion is very short-lived and does not move from the interfacial region to the bulk organic phase, making hydrolysis the dominant reaction pathway. However, the reaction of chlorodifluoromethane with concentrated KOH in dioxane in the presence of tetraphenylarsonium chloride as the catalyst, provided low yields (<30%) of the cyclopropanation products [9]. Therefore, another modified method was developed, especially as this method was limited to nucleophilic alkenes.

**Scheme 5:**
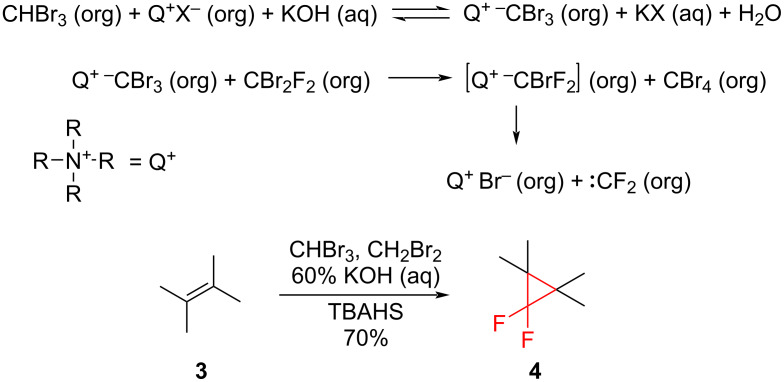
Generation of difluorocarbene in a catalytic two-phase system and its addition to tetramethylethylene (**3**).

It is possible to obtain difluorocarbene from the reaction of bromoform (or methylene bromide) with dibromodifluoromethane. Here, bromoform (or methylene bromide) is deprotonated, resulting in the formation of tribromo- or dibromomethyl carbanions. The so-obtained carbanions form lipophilic ion pairs with the catalyst cation and move into the organic phase, where they react with dibromodifluoromethane. Consequently, carbon tetrabromide (or bromoform) and the ion pair CBrF_2_^−^N^+^Bu_4_ are formed. The ion pair decomposes into TBAB and difluorocarbene, which then can react with alkenes producing the *gem*-difluorocyclopropane derivatives such as **4** (Scheme 5) [21].

**Scheme 6:**
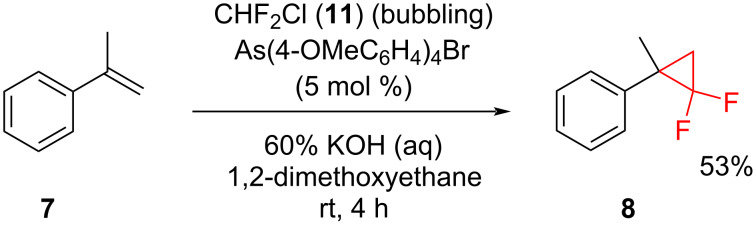
The reaction of methylstyrene **7** with chlorodifluoromethane (**11**) in the presence of a tetraarylarsonium catalyst.

**Chlorodifluoromethane as a source of difluorocarbene in the reaction:** The advantage of using of tetraarylarsonium salts as effective phase-transfer catalysts for the two-phase reaction of chlorodifluoromethane (freon 22, **11**) with α-methylstyrene (**7**) was demonstrated by Barbasiewicz [22] (Scheme 6). The reaction proceeded at room temperature for 4 h with the formation of the cyclopropane derivative **8**.

**Scheme 7:**
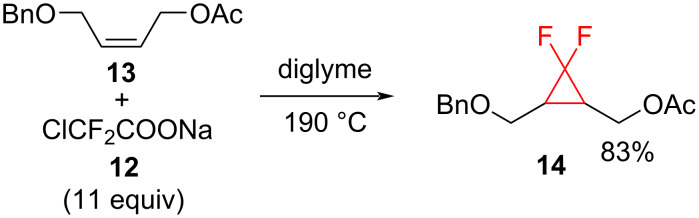
Pyrolysis of sodium chlorodifluoroacetate (**12**) in refluxing diglyme in the presence of alkene **13**.

**Chloro- and bromodifluoroacetate salts as difluorocarbene sources:** The sodium salt of chlorodifluoroacetic acid (ClCF_2_COONa, **12**) is one of the most commonly used reagents for the difluorocyclopropanation. The first published method for the generation of *gem-*difluorocyclopropanes comprised the addition of sodium chlorodifluoroacetate (**12**) to the disubstituted alkene **13** in refluxing diglyme or triglyme at 190 °C (Scheme 7) [23].

**Scheme 8:**
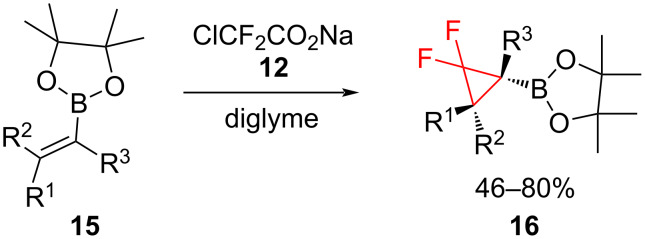
Synthesis of boron-substituted *gem*-difluorocyclopropanes **16**.

The method has been widely used for the difluorocyclopropanation of allylic alcohol derivatives [24], steroids [25], and *N*-Boc-protected enamides [26]. Boron-substituted difluorocyclopropanes **16** can be also obtained from **12**. Fujioka and Amii [27] prepared the versatile building blocks **16** by the reaction of **12** with alkenyl boronates **15** (Scheme 8).

**Scheme 9:**
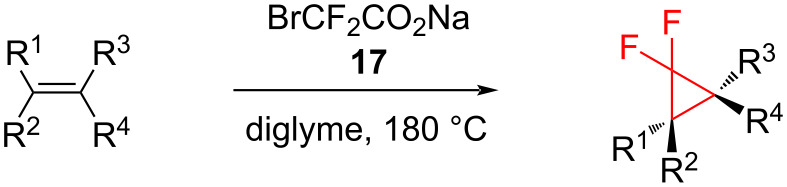
Addition of sodium bromodifluoroacetate (**17**) to alkenes.

Although this method is one of the most popular and reliable ones, it does have some drawbacks, particularly the high temperatures that are required (180–190 °C). Another disadvantage is the use of excess amounts of ClCF_2_COONa (**12**). Thus, the reaction of 2,2-difluorostyrenes and **12** in diglyme at 180 °C gave 1-aryl-2,2,3,3-tetrafluorocyclopropane as a primary product. After prolonged reaction under these conditions, 1,1,2,2-tetrafluoroindanes were the only products isolated [28]. In addition, it is hard to work with sodium chlorodifluoroacetate, as it is highly hygroscopic and deliquescent [29]. Hence, in order to avoid these issues, sodium bromodifluoroacetate (**17**) may be used (Scheme 9).

Amii and co-workers compared the efficiency of the two reagents, ClCF_2_COONa (**12**) and BrCF_2_COONa (**17**), in the difluorocyclopropanation of 1,1-diphenylethene (**18**) [29] and the results are summarized in Table 1. They showed that it was easier and more efficient to work with sodium bromodifluoroacetate (**17**). The application of the same conditions resulted in almost 100% yield, when using **17**. The major advantages of **17** over **12** are that the bromo derivative **17** is stable at room temperature and requires a lower temperature than **12** to decompose to difluorocarbene.

**Table 1:** Comparison of halodifluoroacetates **12** and **17** in the difluorocyclopropanation of 1,1-diphenylethene (**18**).

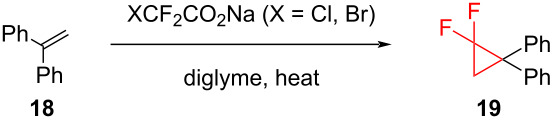			

entry	XCF_2_CO_2_Na	conditions	yield **19** (%)

1	ClCF_2_CO_2_Na (**12**)	180 °C, 20 min	96
2	ClCF_2_CO_2_Na (**12**)	150 °C, 20 min	64
3	BrCF_2_CO_2_Na (**17**)	150 °C, 20 min	99
4	BrCF_2_CO_2_Na (**17**)	120 °C, 20 min	76

**Scheme 10:**
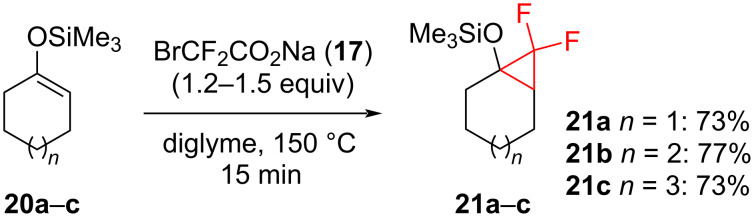
Addition of sodium bromodifluoroacetate (**17**) to silyloxy-substituted cyclopropanes **20**.

By the use of BrCF_2_COONa in diglyme at 150 °C, various alkyl- and aryl-substituted alkenes, allyl alcohol esters, α,β-unsaturated esters, and alkenyl (pinacol) boranes **16** were transformed into the corresponding difluorocyclopropanes in 93–99% yields. Highly sensitive substrates such as trimethylsilylenol ethers **20** can also be used in this method in order to prepare the difluorocyclopropanes **21** with good yields (Scheme 10) [29–30].

In addition, another modification was made in order to increase the speed of the reaction of sodium halodifluoroacetate and alkenes. This was achieved by the use of microwave irradiation in THF solution, which allowed the reactions to be completed within 5 minutes [31].

**Scheme 11:**
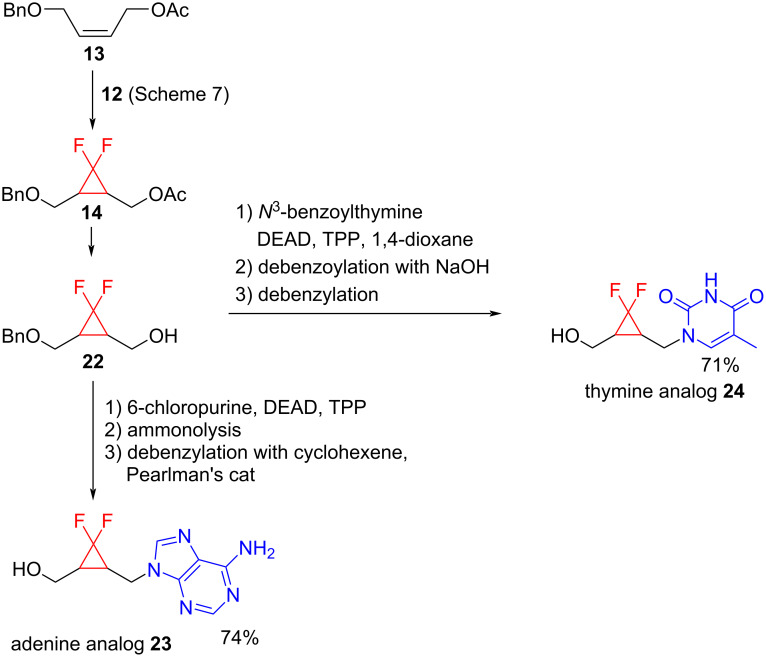
Synthesis of difluorinated nucleosides.

An application of this method to targets of biological interest was provided by Csuk and Eversmann [32] who performed the synthesis of difluorinated nucleosides (Scheme 11). The difluorocyclopropane derivative **14** was prepared using sodium chlorodifluoroacetate (**12**) as a source of the carbene (Scheme 11). The subsequent deacetylation of **14** resulted in the formation of alcohol **22**, which was then reacted with nucleoside analogs via a Mitsunobu reaction to generate the racemic difluorinated carbocyclic homonucleoside analogs **23** and **24** in good yields.

**(Triphenylphosphonio)difluoroacetate (PDFA, Ph_3_P^+^CF_2_CO_2_^−^**) **as a difluorocarbene source:** PDFA is available from the reaction of triphenylphosphine with halodifluoroacetate salts such as BrCF_2_CO_2_K. It exists as a free-flowing white solid that is not sensitive to air or moisture [33]. Upon heating to 80 °C in *N*-methylpyrrolidone, the compound decarboxylates and acts as a source of the ylide Ph_3_P^+^CF_2_^−^, which was used for the Wittig olefination of aldehydes and ketones. However, heating PDFA in nonpolar solvents (e.g., xylene at 90 °C) favors the dissociation of the ylide to release difluorocarbene which is able to effect the cyclopropanation of alkenes [34].

**Scheme 12:**
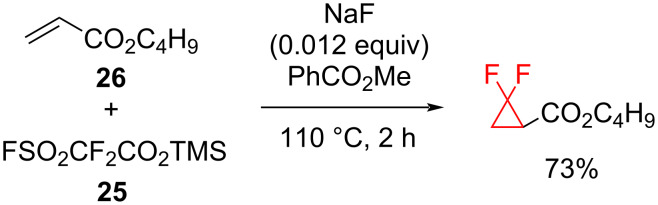
Addition of butyl acrylate (**26**) to difluorocarbene generated from TFDA (**25**).

**Trimethylsilyl fluorosulfonyldifluoroacetate (TFDA) as a difluorocarbene source:** Highly efficient methods for the difluorocyclopropanation of both electron-rich and electron-deficient alkenes using FSO_2_CF_2_COOSiMe_3_ (TFDA, **25**) as a source of difluorocarbene were described by the Dolbier group in 2000 [13,35]. The difluorocarbene generated by this method was able to add at moderate temperatures to unreactive alkenes such as butyl acrylate (**26**) (Scheme 12). Fluoride ions can initiate a chain process, whereby TFDA undergoes desilylation which is followed by a subsequent decarboxylation, and loss of SO_2_ to form difluorocarbene :CF_2_ and F**^−^**; NaF was found to be superior to both CsF and KF as an initiator.

**Scheme 13:**
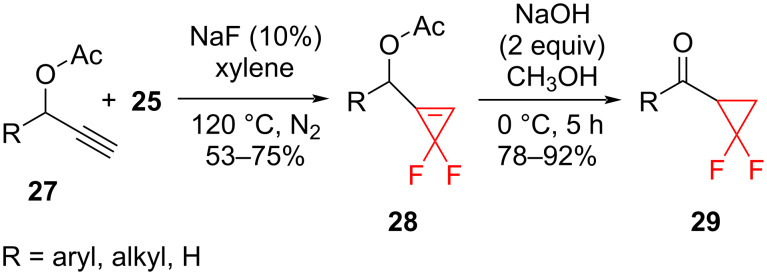
Addition of difluorocarbene to propargyl esters **27** and conversion of the difluorocyclopropenes **28** to difluorocyclopropyl ketones **29**.

Difluorocarbene generated from TFDA (**25**) also readily reacted with propargyl esters **27** at the triple bond (Scheme 13). The difluorocyclopropenes **28** were further converted into the difluorocyclopropyl ketones **29** by alkaline hydrolysis and isomerization [36].

Several nitrogen nucleophiles have been evaluated as catalysts to promote the difluorocarbene formation from TFDA in order to bring about the cyclopropanation of a 2-siloxybuta-1,3-diene derivative; 1,8-bis(dimethylamino)naphthalene (proton sponge) was found to be particularly effective [37].

**Scheme 14:**
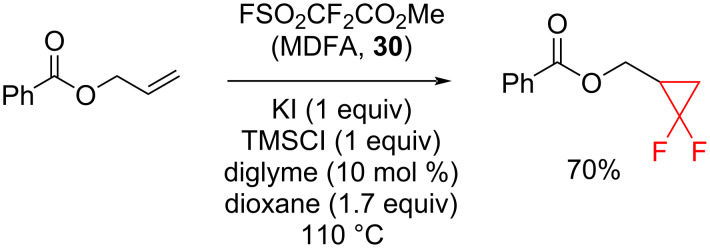
The generation of difluorocyclopropanes using MDFA **30**.

**Methyl 2,2-difluorosulfonyldifluoroacetate as a source of difluorocarbene:** Eusterwiemann et al. devised a method for the generation of difluorocyclopropanes using methyl 2,2-difluoro-2-(fluorosulfonyl)acetate (MDFA, **30**) as a source of difluorocarbene (Scheme 14) [38]. The difluorocyclopropanation of α-methylstyrene (**7**) by MDFA gave the corresponding difluorocyclopropane **8** in 82% NMR yield.

The conditions used with MDFA were similar to those for TFDA. Minimal amounts of solvent were applied, keeping the concentrations high. The fluoride trap TMSCl which is both corrosive and volatile, could be replaced by hexamethyldisiloxane (HMDSO), however, then, the reaction required a longer time to complete. When HMDSO was used in the cyclopropanation of **7** the yield of **8** was decreased to 73% [38]. TFDA (**25**) and MDFA (**30**) have comparable reactivity; however, **30** is a better choice of difluorocarbene source in terms of safety, cost, preparation, and ease of storage.

**Scheme 15:**
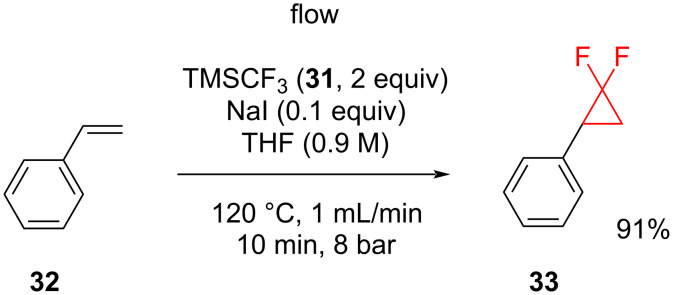
*gem*-Difluorocyclopropanation of styrene (**32**) using difluorocarbene generated from TMSCF_3_ (**31**) under flow conditions.

**The generation of difluorocarbene from trimethyl(trifluoromethyl)silane:** One more modified method, which also increases the rate of the reaction, is the generation of difluorocarbene from TMSCF_3_ (**31**), which is also known as the Ruppert–Prakash reagent [39]. The advantages of this reagent are its safety, low cost, and commercial availability. The reagent is compatible with a range of functionalized substrates for the *gem*-difluorocyclopropanation when using NaI as an initiator (Table 2). Both, electron-rich and comparatively electron-poor examples have been described. Flow reaction conditions were also applied to this reaction (Scheme 15). The reagents were premixed in THF at room temperature and injected into a heated reactor fitted with a back pressure regulator to allow operation at temperatures that exceeded the boiling point of the solvent. In this flow chemistry setup there was an opportunity to control the temperature, pressure, and to make the heat transfer more efficient [40]. The separate injection of a solution of the carbene precursor and of the alkene premixed with the activator did not lead to any improvement. Rullière et al. also tested this method on simple alkenes, electron-rich styrenes, and styrenes with electron-withdrawing substituents in the structure [40]. The yields of the *gem*-difluorocyclopropanes from the styrene derivatives were almost all excellent. On the other hand, simple alkenes gave lower yields.

The reaction of various functionalized alkenes with the CF_3_SiMe_3_–NaI system has been studied (Table 2). Difluorocarbene addition to α-fluorostyrenes enabled the efficient synthesis of trifluorocyclopropanes **34** [41]. The difluorocyclopropanation of protected cyclohexenone yielded cyclopropane **35** [42]. Difluorocyclopropane **36** was formed in high yield from the α-bromopyridine-substituted *N*-Boc-3,4-dehydropiperidine. When the same reaction was attempted on the bromine-free analog, the yield was only 22% [43]. The difluorocyclopropanation of an alkenyl trifluoroborate using the TMSCF_3_–NaI system afforded the boronate derivative **37** [44]. The reagent was also used for the synthesis of organic spiro compounds, containing selectively fluorinated cyclopropanes **38a**,**b** [45], for the preparation of 6,6-difluoro-3-azabicyclo[3.1.0]hexane (**39**) (on a 10 g scale) [46], and of the epothilone B analog **40** [47] (Table 2).

**Table 2:** The synthesis of *gem*-difluorocyclopropanes using TMSCF_3_ (**31**) as the carbene source in combination with sodium iodide as initiator.

entry	substrate	reagents and conditions	compound (yield)	reference

1	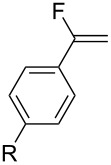 R = H, Br, F, Ph, OMe, NO_2_	TMSCF_3_ (**31**) NaI THF 55 °C, 20 h	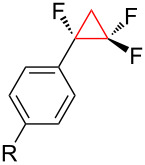 **34** (53–93%)	[41]
2	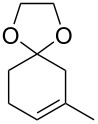	TMSCF_3_ (**31**, 2.5 equiv) NaI (0.5 equiv) THF 65 °C , 12 h	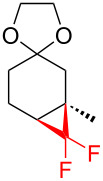 **35** (86%)	[42]
3	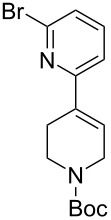	TMSCF_3_ (**31**, 3 equiv) NaI (0.3 equiv) THF 65 °C , 17 h	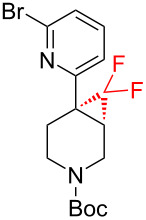 **36** (78%)	[43]
4	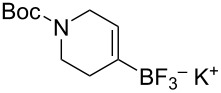	TMSCF_3_ (**31**, 5 equiv) NaI (0.4 equiv) THF 65 °C, 30 min	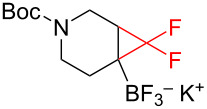 **37** (70%)	[44]
5	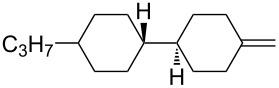	TMSCF_3_ (**31**, 5 equiv) NaI (0.2 equiv) THF 60 °C, 5 h	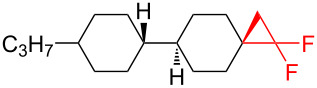 **38a** 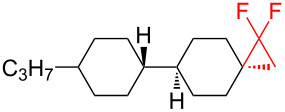 **38b** (a:b ratio, 5:1)	[45]
6	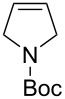	1) TMSCF_3_ (**31**, 2.5 equiv), NaI (0.5 equiv) THF, 65 °C, 4 h 2) MeOH, HCl, 0 °C	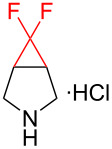 **39** (64%)	[46]
7	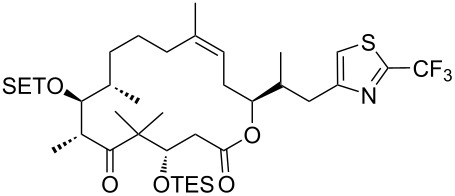	TMSCF_3_ (**31**, 5 equiv) NaI (0.2 equiv) THF 60 °C, 2 h sealed tube	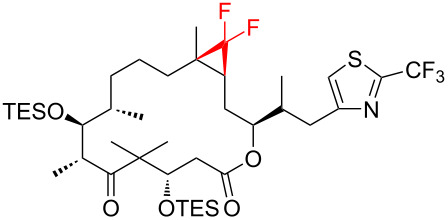 **40** (83%)	[47]

The reagents (chlorodifluoromethyl)trimethylsilane (ClCF_2_SiMe_3_ [48]) and (bromodifluoromethyl)trimethylsilane (ClCF_2_SiMe_3_ [49]) have both been used for the difluorocyclopropanation and gave good yields in reactions with electron-rich alkenes. The formation of difluorocarbene was effected by heating the precursors in the presence of catalytic amounts of halide sources (e.g., tetramethylammonium chloride or tetrabutylammonium bromide). Compared with the difluoromethylenation protocols using TFDA (**25**), MDFA (**30**), or TMSCF_3_ (**31**), the application of BrCF_2_SiMe_3_ has been claimed to be safer and more convenient for large-scale application because of the avoidance of gaseous byproducts [49]. Other mild sources of difluorocarbene include trifluoro(trifluoromethyl)silane (CF_3_SiF_3_ [50]) and difluorotris(trifluoromethyl)phosphorane ((CF_3_)_3_PF_2_ [51]).

**Scheme 16:**
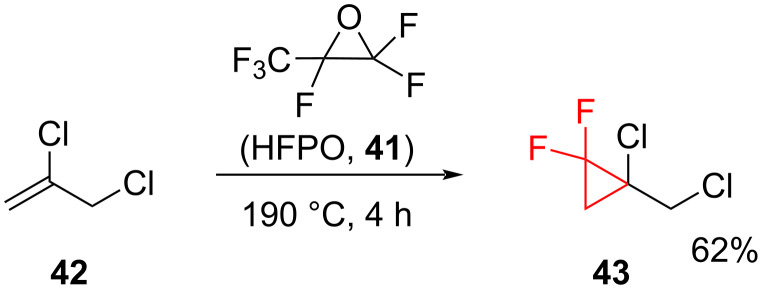
Synthesis of a *gem-*difluorocyclopropane derivative using HFPO (**41**) as a source of difluorocarbene.

**Difluorocarbene generation through the decomposition of hexafluoropropylene oxide upon heating:** Hexafluoropropylene oxide (HFPO, **41**) is an effective and cheap reagent for the difluorocyclopropanation of simple alkyl- and aryl-substituted alkenes [52]. It undergoes decomposition to form difluorocarbene (Scheme 16) at temperatures above 170 °C either under autoclave conditions or by gas-phase co-pyrolysis [53].

**Scheme 17:**
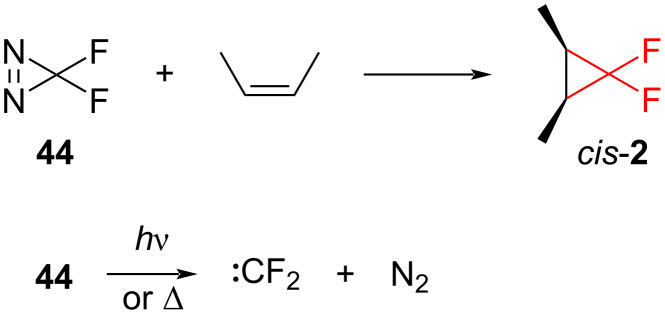
Cyclopropanation of (*Z*)-2-butene in the presence of difluorodiazirine (**44**).

**Photolytic generation of difluorocarbene:** Difluorodiazirine (**44**) is a convenient photochemical source of difluorocarbene (Scheme 17). The compound readily produces difluorocarbene upon photolysis. N_2_ is the leaving group and it is good for LFP studies [54].

Furthermore, pyrolysis is also suitable for difluorocarbene generation from this reagent. Consequently, difluorocarbene is generated, when diazirine **44** is heated above 165 °C. Moreover, the reactions using **44** as a carbene source produce the difluorocyclopropanes in good yields [55]. As for the disadvantages, difluorodiazirine (**44**) is quite explosive.

#### 1.2 Difluorocarbene methods with organometallic sources

**Scheme 18:**
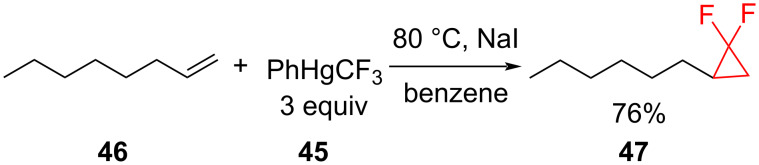
The cyclopropanation of 1-octene (**46**) using Seyferth's reagent (**45**) as a source of difluorocarbene.

**Decomposition of phenyl(trifluoromethyl)mercury in the presence of sodium iodide:** The preparation of difluorocyclopropanes using phenyl(trifluoromethyl)mercury (PhHgCF_3_, **45**, Seyferth's reagent) as a source of difluorocarbene, results in good yields of the products from both electron-rich and electron-poor alkenes [56]. The required decomposition of PhHgCF_3_ (**45**) can be achieved by refluxing in benzene in the presence of NaI (Scheme 18).

In addition to **45**, two other organomercury compounds which have been shown to act as sources of difluorocarbene are iodo(trifluoromethyl)mercury (IHgCF_3_) and bis(trifluoromethyl)mercury (Hg(CF_3_)_2_) [57]. However, despite good synthetic conversions having been obtained with Seyferth's reagent [58] and the general insensitivity of organomercurials to air and moisture, the presence of mercury in all of these structures is a major drawback because organomercury compounds are extremely toxic and environmentally persistent.

**Scheme 19:**
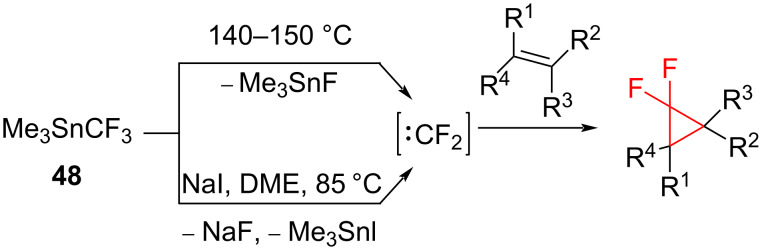
Alternative approaches for the difluorocarbene synthesis from trimethyl(trifluoromethyl)tin (**48**).

**Decomposition of trimethyl(trifluoromethyl)tin in the presence of sodium iodide:** It is also possible to prepare difluorocyclopropanes from olefins and trifluoromethyl derivatives of tin such as trimethyl(trifluoromethyl)tin (**48**). There are two possible ways to obtain difluorocarbene from **48**: thermal (at 140–150 °C, 20–44 h) [59] and iodide ion induced (at 85 °C, 16 h) (Scheme 19) [60]. The trapping with alkenes gave the expected cyclopropanes.

**Scheme 20:**
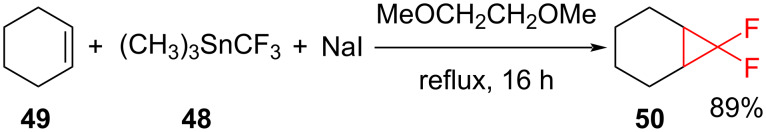
Difluorocyclopropanation of cyclohexene (**49**).

The reaction of (СН_3_)_3_SnСF_3_ (**48**) with NaI (1 equiv) occurred in 1,2-dimethoxyethane (Scheme 20) [60]. The difluorocarbene then added to cyclohexene (**49**) to form difluoronorcarane **50** with good yield. Under similar conditions tetramethylethylene afforded 1,1-difluorotetramethylcyclopropane (**4**).

**Scheme 21:**
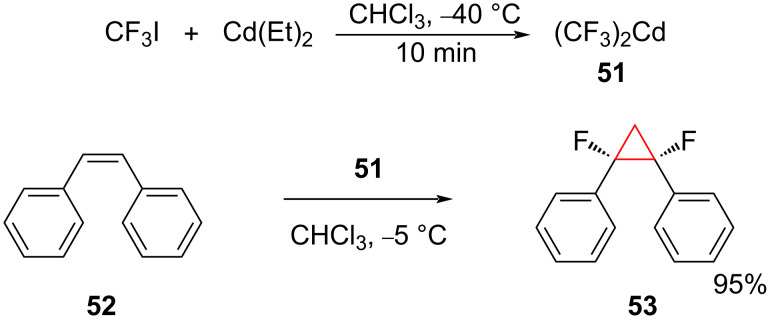
Synthesis of difluorocyclopropane derivative **53** using bis(trifluoromethyl)cadmium (**51**) as the difluorocarbene source.

**Trifluoromethyl derivatives of cadmium, bismuth, zinc, and gold:** Bis(trifluoromethyl)cadmium (**51**) precipitates quantitatively as a white powder from the reaction of CF_3_I with Cd(Et)_2_ (molar ratio 2.5:1) (Scheme 21) in chloroform at −40 °C. A warming to −5 °C was sufficient to liberate difluorocarbene, which was trapped by the addition to alkenes. Thus, *cis*-stilbene (**52**) gave the *gem-*difluorocyclopropane derivative **53** plus cadmium fluoride [61–62]. However, cadmium compounds are highly toxic and furthermore (CF_3_)_2_Cd is pyrophoric in air and liable to explode upon warming to room temperature.

**Scheme 22:**
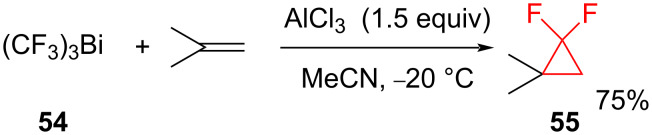
Addition of difluorocarbene generated from tris(trifluoromethyl)bismuth (**54**).

Tris(trifluoromethyl)bismuth (**54**) is also a source of difluorocarbene, that is generated during the reaction of **54** with AlCl_3_ and an alkene at −20 °C (Scheme 22). The difluorocyclopropane **55** was obtained in 75% yield [63].

**Scheme 23:**
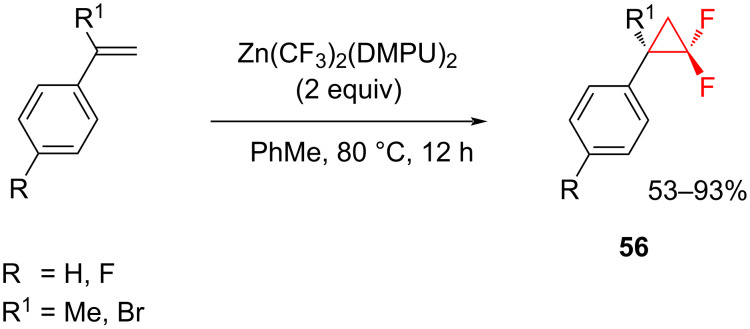
Addition of a stable (trifluoromethyl)zinc reagent to styrenes.

A bis(trifluoromethyl)zinc reagent was employed as the difluorocarbene source for the *gem*-difluorocyclopropanation of alkenes or alkynes via thermal decomposition [64]. The reagent was generated from trifluoromethyl iodide (CF_3_I) and Zn dust (or ZnEt_2_) in 1,3-dimethyl-3,4,5,6-tetrahydro-2(1*H*)-pyrimidinone (DMPU) [65] and later isolated [66]. The reaction of Zn(CF_3_)_2_(DMPU)_2_ (2 equiv) with styrenes proceeded efficiently in toluene to provide the difluorocyclopropanes **56** in 53–93% yields (Scheme 23) [64].

Fürstner et al. [67] showed that (trifluoromethyl)gold(I)triphenylphosphine in dichloromethane can be used for the production of difluorocyclopropanes at low temperatures. The advantage of the method is its stereoselectivity. The disadvantages include the stoichiometric use of gold, low temperatures, process length (17 hours), and the low yields of products (12–45%).

#### 1.3 Non-carbene methods

**Scheme 24:**
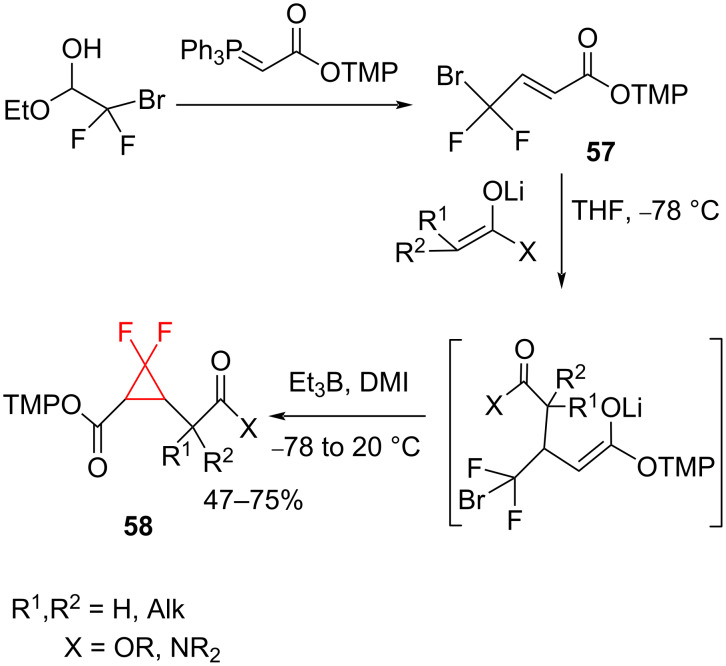
The preparation of 2,2-difluorocyclopropanecarboxylic acids of type **58**.

Although the generation of difluorocyclopropanes often involved difluorocarbene, several non-carbene methods have also been developed. Taguchi and Okada developed a protocol for the preparation of 2,2-difluorocyclopropanecarboxylic acid derivatives **58** by the Michael addition of ester and amide enolates to 2,4,6-trimethylphenyl 4-bromo-4,4-difluorocrotonate (**57**) followed by an Et_3_B-initiated radical cyclization (Scheme 24) [68].

**Scheme 25:**
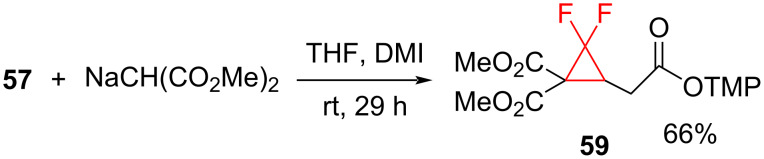
Difluorocyclopropanation via Michael cyclization.

Furthermore, when the sodium salt of dimethyl malonate was used as the Michael donor the cyclopropane formation did not require Et_3_B (Scheme 25).

**Scheme 26:**
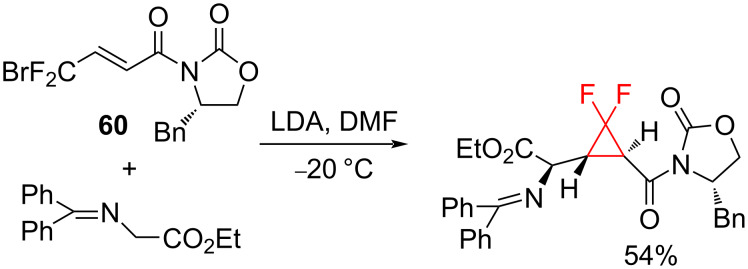
Difluorocyclopropanation using *N*-acylimidazolidinone **60**.

The work was extended to include boron-free, diastereoselective versions incorporating *N*-acylimidazolidinone chiral auxiliaries (Scheme 26).

**Scheme 27:**
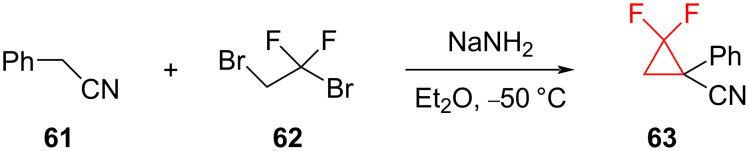
Difluorocyclopropanation through the cyclization of phenylacetonitrile (**61**) and 1,2-dibromo-1,1-difluoroethane (**62**).

**Cyclization reaction of phenylacetonitrile and 1,2-dibromo-1,1-difluoroethane:** Kagabu et al. showed that the nitrile **63** could be obtained by the reaction of phenylacetonitrile (**61**) with 1,2-dibromo-1,1-difluoroethane (**62**) using sodium amide as the base (Scheme 27) [69]. However, the yield of this reaction was only 10%.

**Scheme 28:**
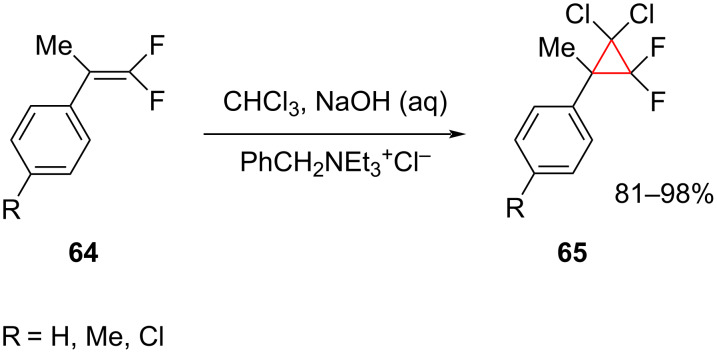
*gem*-Difluoroolefins **64** for the synthesis of functionalized cyclopropanes **65**.

The *gem*-difluorocyclopropanes **65** were synthesized from the reaction of *gem*-difluoroolefins **64** and chloroform in an aqueous 40% NaOH solution using the phase-transfer catalyst benzyltriethylammonium chloride (Scheme 28) [70–71]. Although difluorocarbene is not involved in the cyclopropanation step, this approach does employ dichlorocarbene.

#### 1.4 Transformation of functional groups

**Scheme 29:**
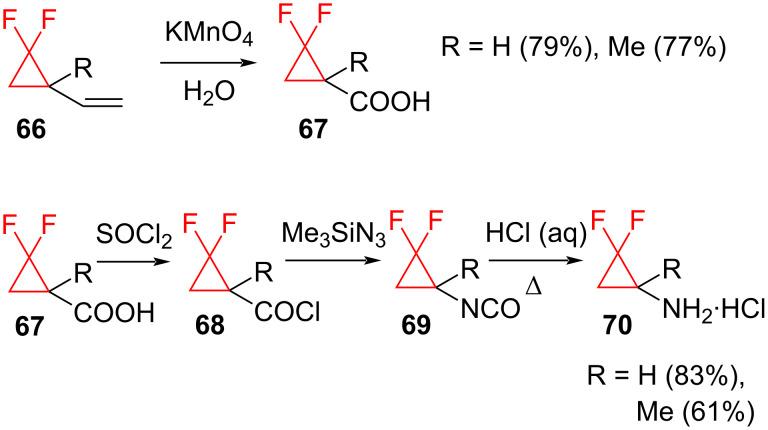
Preparation of aminocyclopropanes **70**.

*gem-*Difluorocyclopropanes easily undergo various transformations leading to the formation of a diversity of useful materials. Although *gem*-difluorocyclopropanes contain a strained ring, they are kinetically stable under the conditions employed for many synthetically important reactions. These include the catalytic hydrogenolysis of benzyl ethers (H_2_, Pd) [72], DIBAL-H reduction of esters to form alcohols [73], oxidative cleavage of vinyl groups to form carboxylic acids (KMnO_4_) [74], and the conversion of the acids into amines using the Curtius rearrangement (SOCl_2_, followed by Me_3_SiN_3_, thermolysis, and acid hydrolysis of the intermediate isocyanate, Scheme 29) [74]. Such transformations proceed with the conservation of the difluorocyclopropane unit and complement the methods for the cyclopropyl-ring synthesis discussed in the previous sections.

**Scheme 30:**
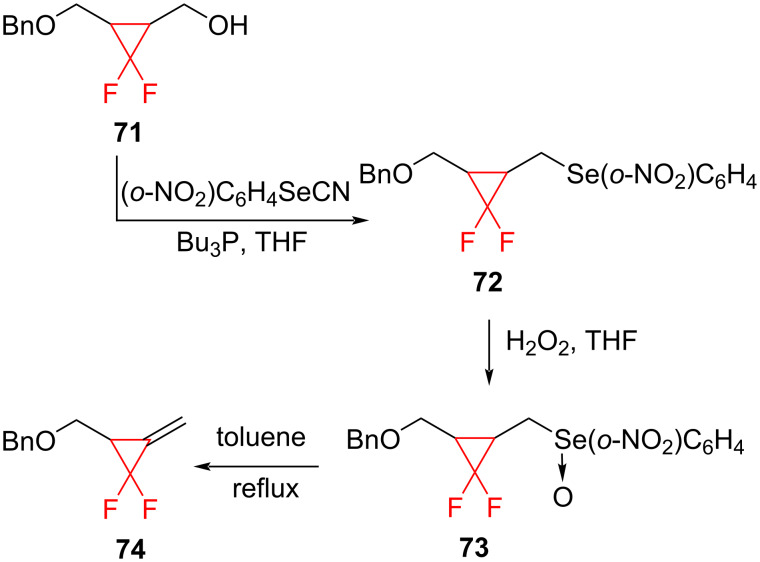
Synthesis of fluorinated methylenecyclopropane **74** via selenoxide elimination.

**Generation of fluorinated methylenecyclopropanes:** Fluorinated methylenecyclopropanes are of interest as Michael acceptors and as substrates for thermal rearrangements. As they are not readily available by difluorocarbene addition to allene derivatives, Taguchi et al. developed an alternative route to these compounds by selenoxide elimination (Scheme 30) [75]. Later, this approach was modified by Wang and co-workers [76].

**Scheme 31:**
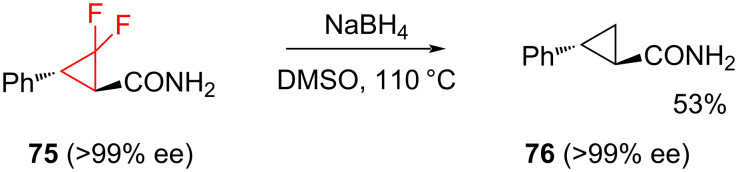
Reductive dehalogenation of (1*R*,3*R*)-**75**.

It is also possible to remove the fluorine substituents from difluorocyclopropanes while preserving the three-membered ring. The reductive defluorination of the difluorocylopropane derivative **75** by the treatment with excess NaBH_4_ in hot DMSO (Scheme 31) gave the corresponding cyclopropane **76** [77]. Caution is advised in view of a recent report that NaBH_4_ lowers the onset temperature for the thermal decomposition of DMSO [78].

The asymmetric difluorocyclopropanation has not yet been developed to the extent achieved for the epoxidation. Consequently, the enantioselective functional group interconversions on prochiral or racemic difluorocyclopropane and difluorocyclopropene derivatives have provided important ways of obtaining enantiomerically pure cyclopropanes. The key reactions in this context are the enzyme-catalyzed formation and hydrolysis of esters and the hydrogenation of difluorocyclopropenes [73,79].

**Enzymatic hydrolysis or esterification:** The first example of the enzymatic resolution of *gem-*difluorocyclopropanes was reported by Itoh et al. [80]. The prochiral diacetate of *cis-*1,2-bis-(hydroxymethyl)-3,3-difluorocyclopropane was converted into the corresponding monoacetate through *Alcaligenes* sp. lipase-catalyzed hydrolysis with >99% enantiomeric excess.

**Scheme 32:**
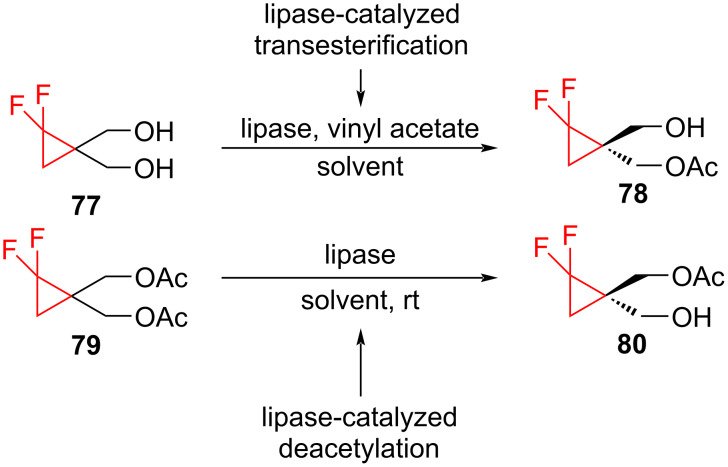
Synthesis of chiral monoacetates by lipase catalysis.

Kirihara et al. have reported the synthesis of the separate enantiomers of 2,2-difluoro-1-aminocyclopropanecarboxylic acid, which are analogs of the naturally occurring 1-aminocyclopropanecarboxylic acid [81]. The authors obtained the chiral monoacetate intermediates (*R*)-**78** and (*S*)-**80** by lipase-catalyzed methods. The lipase-catalyzed asymmetric transesterification of prochiral diol **77** and the deacetylation of the prochiral diacetate **79** resulted in the formation of the (*R*)-monoacetate (*R*)-**78** and (*S*)-monoacetate (*S*)-**80**, respectively (Scheme 32). As for the transesterification, a high yield (96.5%) and enantioselectivity (91.3% ee) were obtained using lipase PS in benzene. In the case of the deacetylation, the use of Amano PS lipase in acetone gave a high yield (86.2%), enantioselectivity (91.7% ee), and smooth hydrolysis.

Wang et al. reported the enantioselective biotransformations of geminally difluorinated cyclopropanecarbonitriles and amides in 2004 [77]. They transformed *gem-*difluorocyclopropane derivatives with the help of a soil microorganism, *Rhodococcus* sp. AJ270, which provided a very effective nitrile hydratase–amidase-containing biocatalytic system and showed a high chemo-, regio-, and enantioselectivity in the hydrolysis of nitriles and dinitriles.

**Scheme 33:**
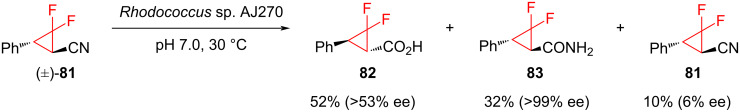
Transformation of (±)-*trans*-**81** using *Rhodococcus* sp. AJ270.

The biocatalytic transformations of nitrile **81** (Scheme 33) supplied an effective route to optically active 2,2-difluorosubstituted 3-phenylcyclopropanecarboxylic acid **82** and amide **83** in both enantiomeric forms (Scheme 33). The biotransformation of the *gem-*difluorocyclopropane **81** produced good results for both the rate and yield. The (1*S*,3*S*)-acid **82** and (1*R*,3*R*)-amide **83** were synthesized in 52% yield with 53% ee and 32% yield with >99% ee, respectively.

**Scheme 34:**
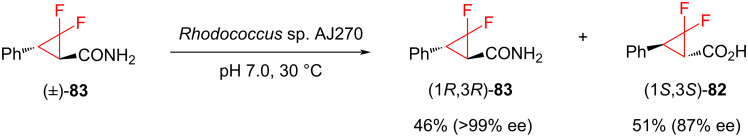
Transformation of (±)-*trans*-**83** using *Rhodococcus* sp. AJ270.

The biotransformation of *gem-*difluorocyclopropanecarboxamide (±)-**83** (Scheme 34) occurred rapidly and under mild conditions to give (1*R*,3*R*)-amide **83** (46% yield, >99% ee) and (1*S*,3*S*)-acid **82** (51% yield, 87 % ee).

**Scheme 35:**
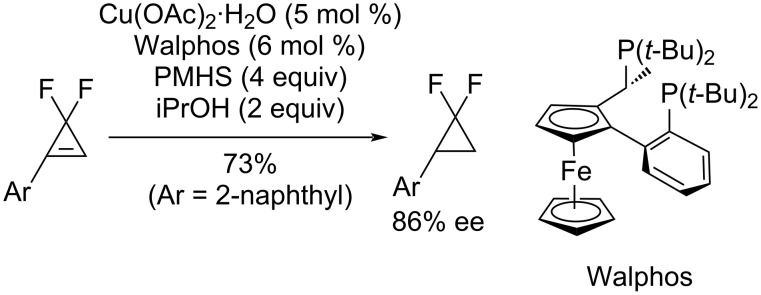
Hydrogenation of difluorocyclopropenes through enantioselective hydrocupration.

**Enantioselective hydrogenation of difluorocyclopropenes:** Recently, Mikami and co-workers reported the enantioselective hydrocupration of difluorocyclopropenes in the presence of chiral diphosphine ligands using stoichiometric hydride sources that included polymethylhydrosiloxane (PMHS) and organoboranes (Scheme 35) [79].

**Scheme 36:**
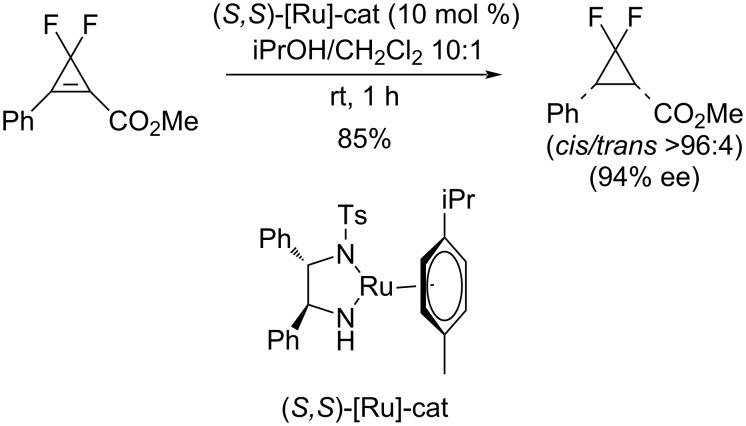
Enantioselective transfer hydrogenation of difluorocyclopropenes with a Ru-based catalyst.

Cossy and co-workers have achieved the catalytic asymmetric transfer hydrogenation with isopropanol as reductant, in conjunction with a Noyori–Ikariya ruthenium-based homogeneous catalyst (Scheme 36) [73].

### 2 Reactions of difluorocyclopropane and its derivatives

Difluorocyclopropanes are synthetically useful substrates for a variety of reactions such as thermal rearrangements, carbocation, carbanion, and radical chemistry. Furthermore, *gem-*difluorocyclopropanes readily go through carbonylation, dehalogenation, and annulation, resulting in various useful materials.

#### 2.1 Thermal rearrangements

The substitution of hydrogen with fluorine in cyclopropane leads to a significant weakening of the C–C bond opposite to the fluorine atom. A consequence of this is the tendency of fluorocyclopropanes, and in particular *gem-*difluorocyclopropanes, to undergo various transformations initiated by a homolytic C–C bond breaking.

**Scheme 37:**
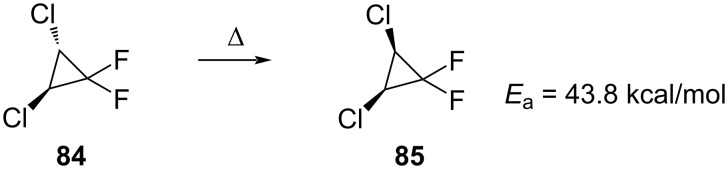
The thermal transformation of *trans*-1,2-dichloro-3,3-difluorocyclopropane (**84**).

**Thermal stereomutation:** In 1975, Staricco and co-workers described the thermal isomerization of *trans*-1,2-dichloro-3,3-difluorocyclopropane (**84**) (Scheme 37) [82].

**Scheme 38:**
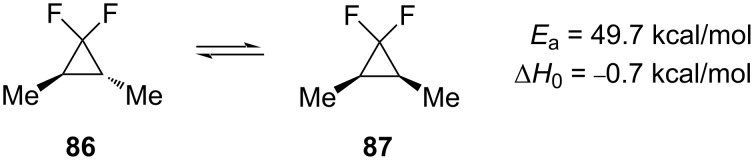
*cis*–*trans*-Epimerization of 1,1-difluoro-2,3-dimethylcyclopropane.

Further research in this area was performed by the groups of Jefford [83] and Dolbier [84], who studied the 1,1-difluoro-2,3-dimethylcyclopropanes **86** and **87** (Scheme 38).

**Scheme 39:**
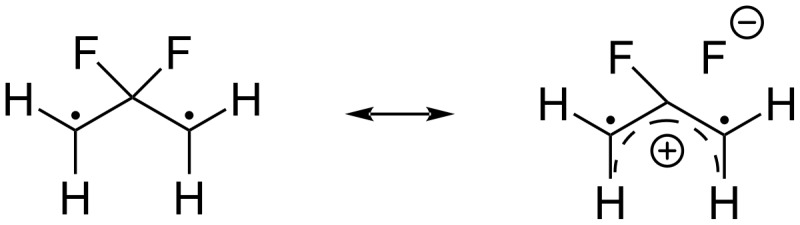
2,2-Difluorotrimethylene diradical intermediate.

Dolbier found that geminal fluorine substituents lowered the activation energies for both *cis*–*trans*-isomerization and for the transformation of vinylcyclopropanes into cyclopentenes. Both processes could occur by a C–C-bond homolysis to form a diradical. Computational studies by Gety, Hrovat, and Borden indicated that there would be a preference for disrotation at C2 and C3 during stereomutation in 1,1-difluorocyclopropanes [85]. An important feature in the fluorinated system was the stabilization of the intermediate 2,2-difluorotrimethylene radicals due to the conjugation of the radical centers with the σ*-orbital of C–F bond, which can be represented by a dipolar resonance structure containing the 2-fluoroallyl cation and fluoride anion (Scheme 39).

**Scheme 40:**
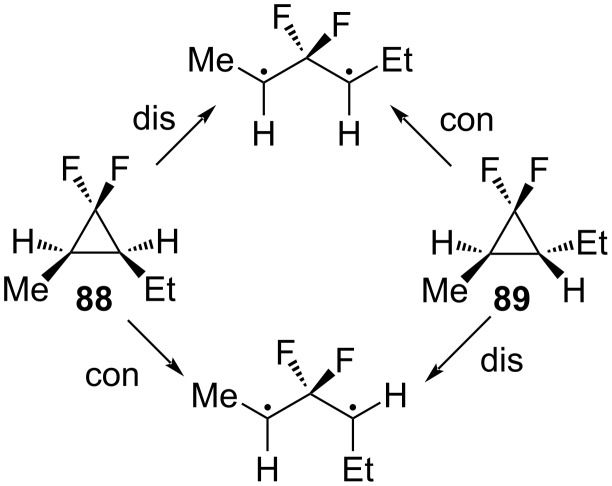
Ring opening of stereoisomers **88** and **89**.

A subsequent comparison of the rates of racemization with those of epimerization confirmed experimentally the preference for coupled disrotatory motions in the opening and closing of 2,3-dialkyl-1,1-difluorocyclopropanes (Scheme 40) [86].

**Vinylcyclopropane rearrangements:** O’Neal and Benson examined the influence of fluorine substituents on the kinetics of the vinylcyclopropane-to-cyclopentene rearrangement [87]. They noted the effect of an additional strain (approximately 5 kcal/mol per fluorine atom) in raising the kinetic reactivity of difluorocyclopropanes and lowering the temperature required for the rearrangement. Furthermore, another effect of the geminal substitution was a weakening of the bond opposite to the CF_2_ fragment by 8–10 kcal/mol.

**Scheme 41:**
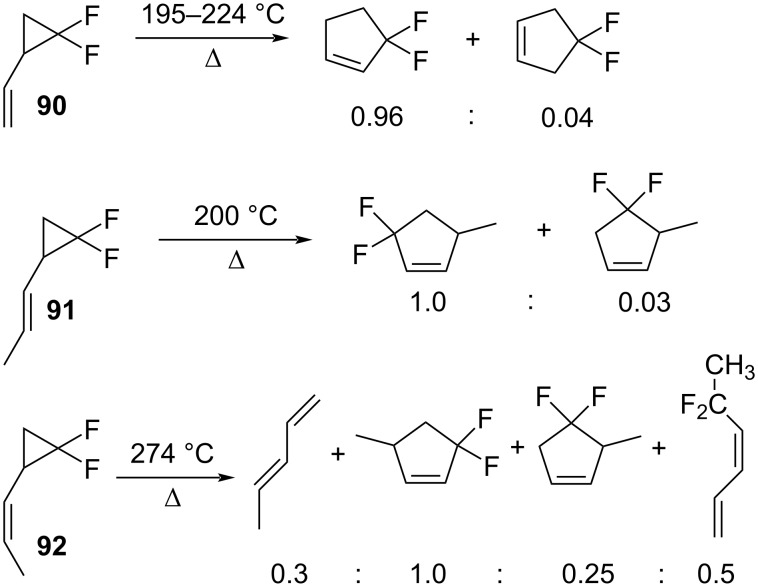
[1,3]-Rearrangement of alkenylcyclopropanes **90**–**92**.

Dolbier et al. studied the thermal rearrangements of 2,2-difluoro-1-alkenylcyclopropanes **90**–**92** (Scheme 41) [88]. All three compounds underwent a highly regioselective cleavage of the C1–C3 bond. Hence, the major products of all rearrangements were produced via [1,3]-sigmatropic shifts (Scheme 41).

**Scheme 42:**
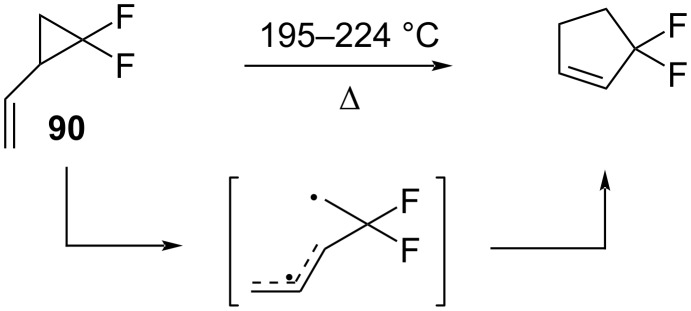
Thermolytic rearrangement of 2,2-difluoro-1-vinylcyclopropane (**90**).

The products were the result of the breaking of the C–C bond opposite to the CF_2_ moiety, which was followed by the recyclization of the intermediate diradical (Scheme 42). The activation energy for the rearrangement of **90** was lower by 9.4 kcal/mol than for the parent hydrocarbon system **92.** The activation energy of the *trans*-isomer **91** was greater than that of *cis*-isomer **91** (>6 kcal/mol), because of the need to attain a cisoid conformation prior to the rearrangement.

**Scheme 43:**
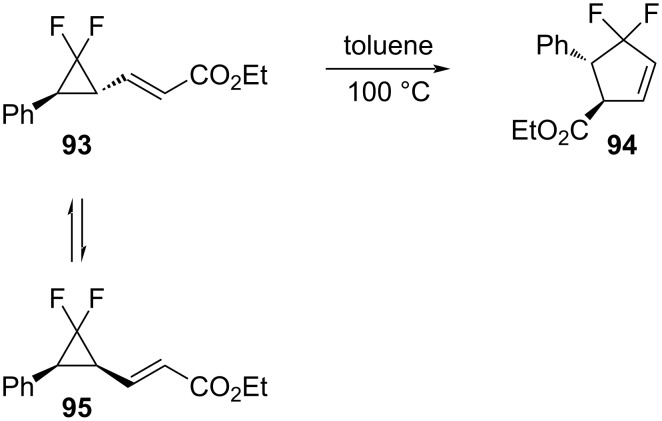
Thermal rearrangement for ethyl 3-(2,2-difluoro)-3-phenylcyclopropyl)acrylates **93** and **95**.

The thermal vinylcyclopropane rearrangement of ethyl *trans*-3-(2,2-difluoro-3-phenylcyclopropyl)acrylate (**93**) proceeded at 100 °C, resulting in the difluorinated cyclopentene **94** with the substituents oriented trans to each other (Scheme 43) [89]. The *cis*-isomer **95** was unable to rearrange directly to a cyclopentene and first isomerized to give **93**. Alkenyldifluorocyclopropanes that were derived from 2-siloxybutadienes underwent analogous rearrangements to afford 1-siloxy-5,5-difluorocyclopentenes [90].

**Scheme 44:**
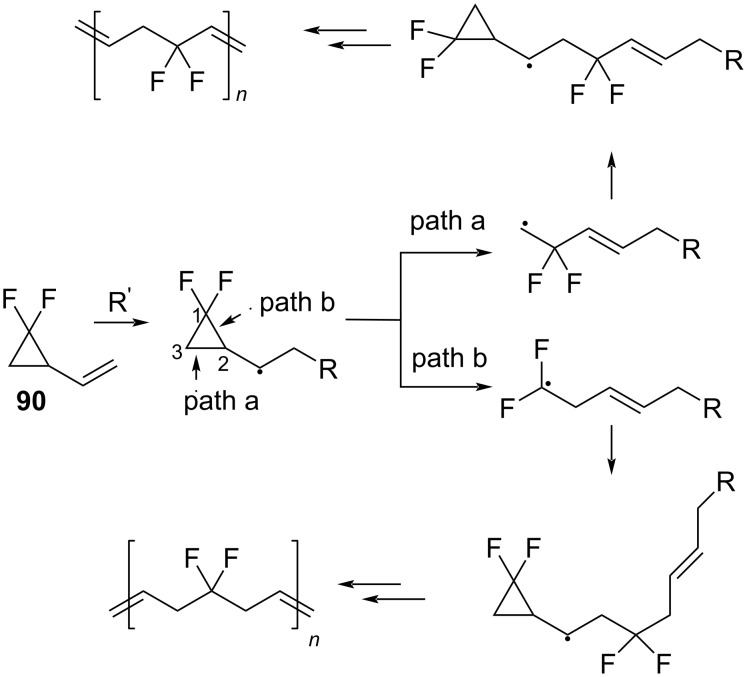
Possible pathways of the ring opening of 1,1-difluoro-2-vinylcyclopropane.

The radical ring-opening polymerization (RROP) provides a synthetic route to fluoropolymers, which are useful materials [91]. The RROP of *gem*-difluorovinylcyclopropane (**90**) gave mainly the polymer with an unsymmetrical repeating unit, by the cleavage of the C2–C3 bond in the ring (Scheme 44, path a). However, 10% of the symmetrical product originating from a C1–C2 bond cleavage (path b) were also observed.

**Methylenecyclopropane rearrangements:** Although *gem-*difluoromethylenecyclopropanes (F_2_MCPs) have poor accessibility, there has been much interest in their thermal rearrangements.

**Scheme 45:**
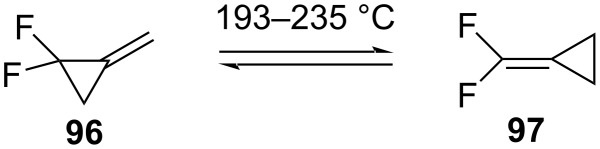
Equilibrium between 1,1-difluoro-2-methylenecyclopropane (**96**) and (difluoromethylene)cyclopropane **97**.

Dolbier examined the rearrangement of 1,1-difluoro-2-methylenecyclopropane (**96**) (Scheme 45) [92]. At 210 °C the rate of cleavage of the proximal bond was only 3.8 times faster than for the analogous hydrocarbon. It was also observed that the equilibrium lay significantly in favor of the rearranged product **97**, which was by 1.9 kcal/mol more stable.

**Scheme 46:**
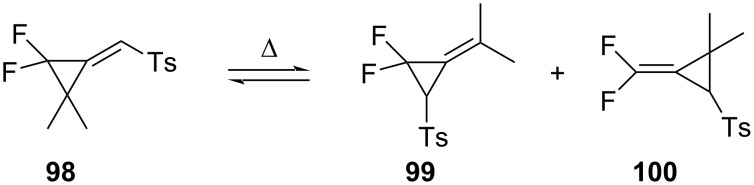
Ring opening of substituted 1,1-difluoro-2,2-dimethyl-3-methylenecyclopropane **98**.

The thermal ring opening of the tosyl-substituted 1,1-difluoro-2,2-dimethyl-3-methylenecyclopropane **98** led to the thermodynamically more stable products **99** and **100**, respectively (Scheme 46) [93].

**Scheme 47:**
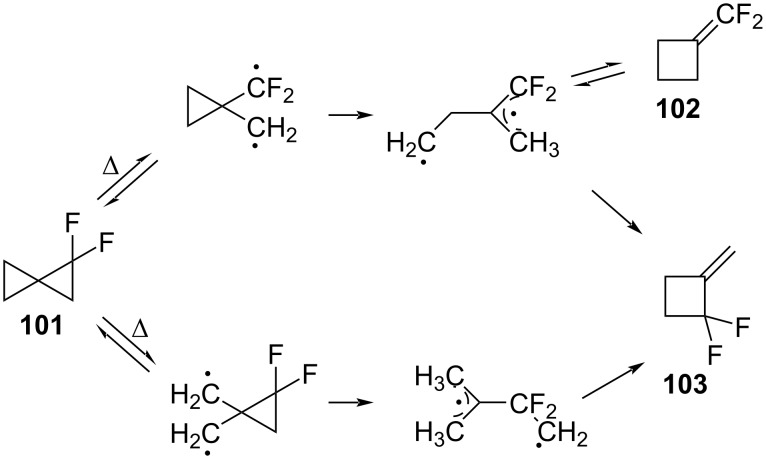
1,1-Difluorospiropentane rearrangement.

**Spiropentane rearrangements:** Gajewsky found that the rearrangement of the hydrocarbon spiropentane to form methylenecyclobutane occurred with the cleavage of the C1–C2 bond [94]. Dolbier then used deuterium labeling to study the analogous reaction of 1,1-difluorospiropentane (**101**) (Scheme 47) [95]. The cleavage of the C1–C2 bond that is proximal to the fluorine resulted in the formation of two isomeric methylenecyclobutane derivatives **102** and **103** by a radical cyclization (Scheme 47). The minor product **102** underwent a fast rearrangement to produce the major product **103**. An alternative pathway, that involved the cleavage of the C4–C5 bond in **101**, also led to the product **103**.

#### 2.2 Ring opening of *gem-*difluorocyclopropanes by external reagents

*gem*-Difluorocyclopropanes have unique properties that arise from the strain of the cyclopropane ring combined with the electronic properties of fluorine. Many of the reactions involve ring-opening processes, the course of which can be controlled by the selection of the reagents and catalysts. These have an influence on the mechanism and regioselectivity of the C–C bond cleavage. Although there are several different mechanisms for ring-opening reactions, in most cases there is a cleavage of the most weakened C–C bond due to the fluorine effect. This C–C bond is opposite to the fluorinated fragment (the distal bond) [2].

**Scheme 48:**
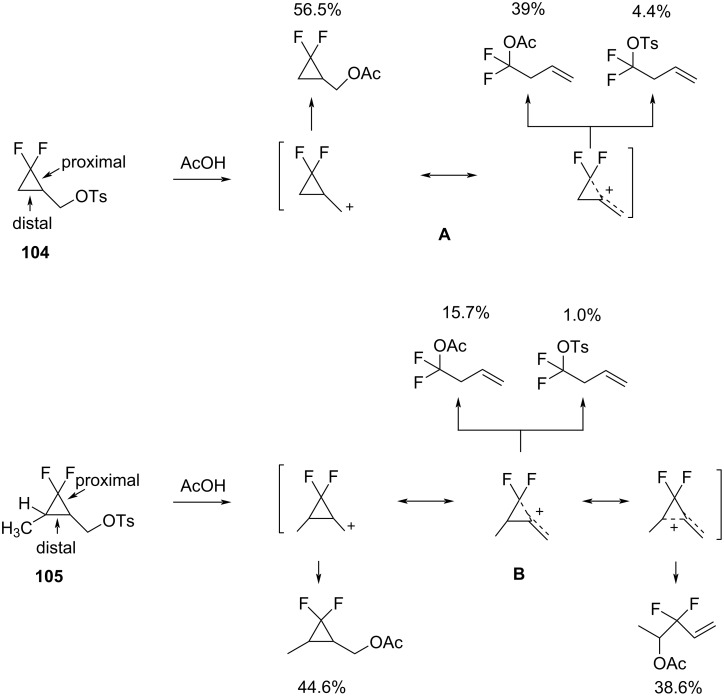
Acetolysis of (2,2-difluorocyclopropyl)methyl tosylate (**104**) and (1,1-difluoro-2-methylcyclopropyl)methyl tosylate (**105**).

**The ring-opening reactions of (2,2-difluorocyclopropyl)methyl systems:** Dolbier investigated the acetolysis of tosylates **104** and **105** (Scheme 48) [96]. The difference between compounds **104** and **105** is the presence of a methyl substituent in **105**, which is associated with a difference in the regioselectivity of the C–C bond cleavage. The dissociation of the tosylate **104** to generate a cyclopropylmethyl carbocation **A** was accompanied by the cleavage of the proximal bond to form homoallylic products. The regioselectivity of the ring opening was attributed to the stabilization of the developing cationic center by the +M effect of the fluorine atoms. The formation of the 2,2-difluorohomoallyl cation or 3,3-difluorocyclobutyl cation did not occur as a result of the strong destabilization by the −I effect of the fluorine atoms [96]. On the other hand, the principal ring-opened product of **105** derives from the cleavage of the distal bond. In this case, the methyl substituent was superior to the two fluorine atoms in stabilizing an adjacent cationic center in **B**. Therefore, the ring opening proceeded via the disruption of the C–C bond opposite to the CF_2_ fragment and the formation of a 2,2-difluorohomoallyl cation.

**Scheme 49:**
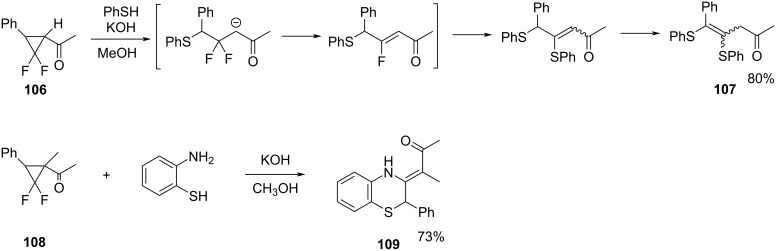
Ring opening of *gem*-difluorocyclopropyl ketones **106** and **108** by thiolate nucleophiles.

**Cleavage of the distal bond. Ring opening of *gem*-difluorocyclopropyl ketones:** The *gem*-difluorocyclopropyl ketones such as **106** and **108** underwent nucleophilic ring-opening reactions induced by thiolate nucleophiles. A distal bond cleavage occurred regioselectively via difluoroenolate intermediates that could participate in subsequent elimination and substitution of fluoride, leading to good yields of the fluorine-free products **107** and **109** (Scheme 49) [97–98].

**Scheme 50:**
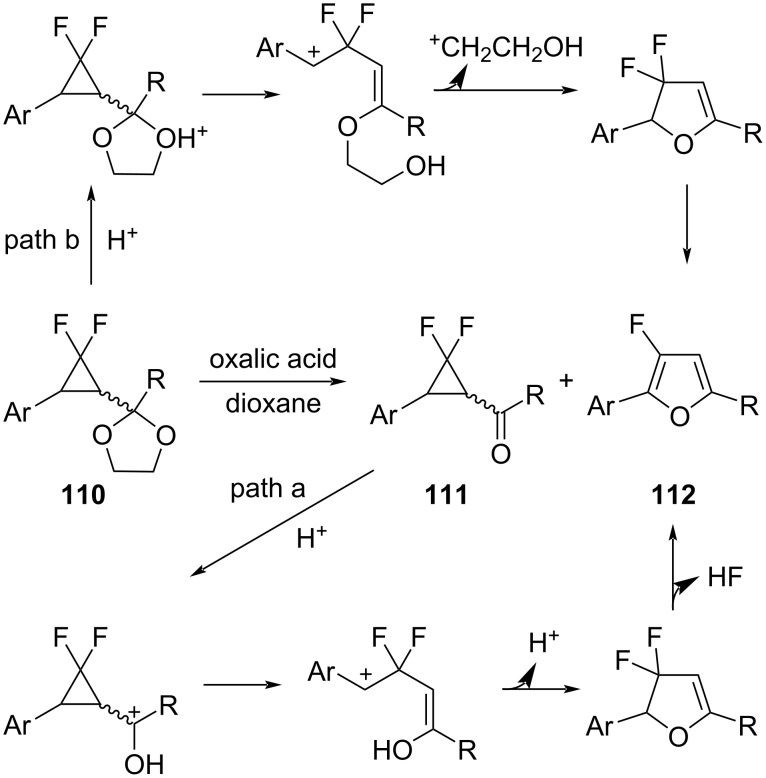
Hydrolysis of *gem-*difluorocyclopropyl acetals **110**.

Xu and Chen studied the acid-catalyzed hydrolysis of *gem*-difluorocyclopropyl acetals **110** to form 2-aryl-3-fluorofurans **112** (Scheme 50) [99]. The reaction could proceed either via the intermediacy of the *gem*-difluorocyclopropyl ketone **111** (path a) or by the direct rearrangement of the protonated acetal (path b). Recently, the group of Amii has reported the conversion of 1-benzoyl-2,2-difluoro-3-phenylcyclopropane and its derivatives into 3-fluoro-2,5-diphenylfuran derivatives following the brief exposure to triflic acid (2 equiv) in cold dichloromethane [100].

**Scheme 51:**
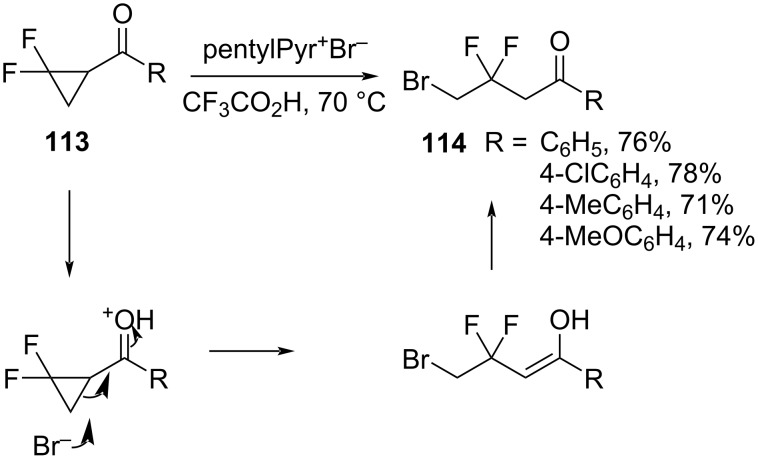
Ring-opening reaction of 2,2-difluorocyclopropyl ketones **113** in the presence of ionic liquid as a surrogate of HBr reagents.

Dolbier et al. described the ring opening of 2,2-difluorocyclopropyl ketones **113** (Scheme 51) [101]. The reactions were mediated by acids and an ionic liquid. 3-Bromo-2,2-difluoropropyl ketones **114** were formed in good to excellent yields by an overall addition of HBr accompanied by a distal bond cleavage.

**Scheme 52:**
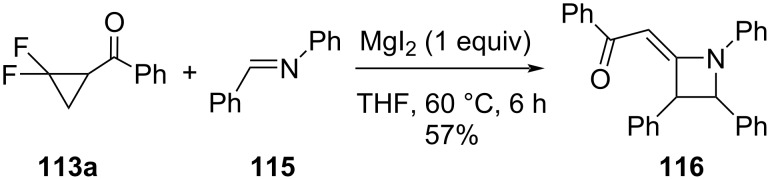
Ring opening of *gem-*difluorocyclopropyl ketones **113a** by MgI_2_-initiated reaction with diarylimines **115**.

Dolbier et al. also studied the MgI_2_-facilitated reactions of aryl-2,2-difluorocyclopropyl ketones **113** with imines **115**, which led to alkylideneazetidines **116** (Scheme 52) [102]. The MgI_2_ acted as a Lewis acid and reducing agent, effecting the distal C–C bond cleavage in **113a** to form an allenyl ketone, or an equivalent fluoro,iodo-enone species, either of which could then have added to the imine **115** and led to the observed product. Only diarylimines were utilized in this study, largely because of their ease of preparation and stability.

**Scheme 53:**
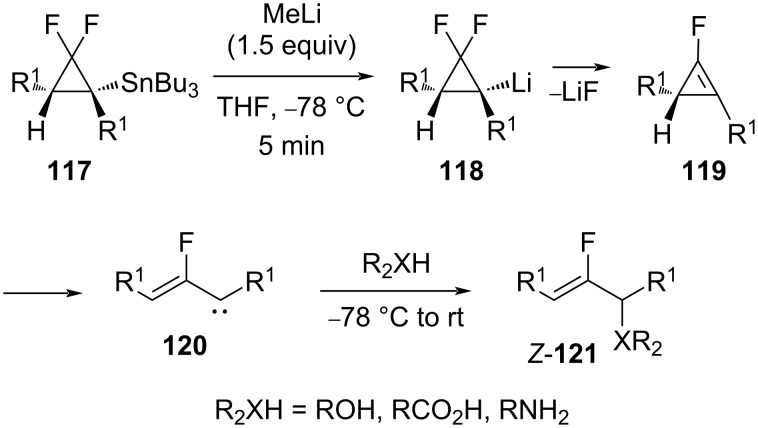
Ring-opening reaction of *gem-*difluorocyclopropylstannanes **117**.

**Ring-opening reaction of *gem*-difluorocyclopropylstannanes:** Konno and co-workers reported the conversion of cyclopropylstannanes **117** into monofluoro derivatives of allylic alcohols, ethers, esters, and amines (**121**, Scheme 53) [103]. They proposed that an initial tin–lithium exchange was followed by a β-elimination of LiF to form the intermediate cyclopropenes **119**. The ring opening of the latter then generated the vinylcarbenes **120**. The carbenes **120** could then insert into the O–H and N–H bonds of water, alcohols, carboxylic acids, and amines to form the observed products **121**.

**Scheme 54:**
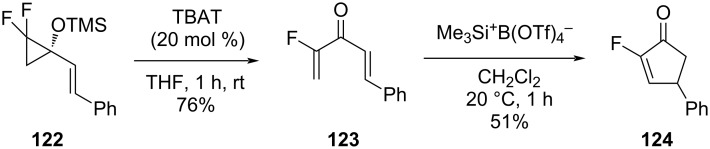
Preparation of 1-fluorovinyl vinyl ketone **123** and the synthesis of 2-fluorocyclopentenone **124**. TBAT = *n-*Bu_4_N^+^ SiF_2_Ph_3_^−^.

1,1-Difluoro-2-siloxy-2-vinylcyclopropane (**122**) was subjected to a fluoride-catalyzed ring opening to afford 1-fluorovinyl vinyl ketones such as **123**. These compounds underwent a Lewis acid-catalyzed Nazarov cyclization with the strong silylating agent Me_3_Si**^+^** B(OTf)_4_^−^ to afford the corresponding 2-fluorocyclopentenone derivatives, e.g., compound **124** (Scheme 54) [37,90].

**Scheme 55:**
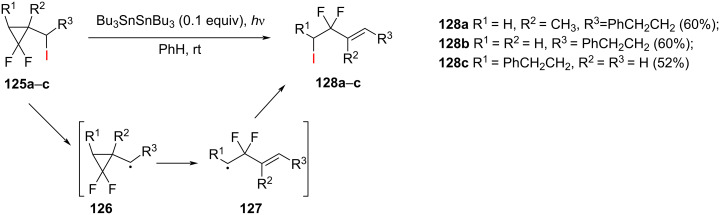
Iodine atom-transfer ring opening of 1,1-difluoro-2-(1-iodoalkyl)cyclopropanes **125a**–**c**.

**Radical-mediated ring-opening reaction:** The photochemical iodine atom-transfer ring opening of 1,1-difluoro-2-(1-iodoalkyl)cyclopropanes **125a**–**c** was initiated by hexabutylditin (Scheme 55) [104]. The (*E*)-difluorohomoallyl iodides **128a**–**c** were isolated in yields ranging from 52 to 60%. The proposed reaction pathway involved the formation of the cyclopropylmethyl radical **126**, which rapidly underwent ring opening to give the homoallyl radical **127**.

**Scheme 56:**
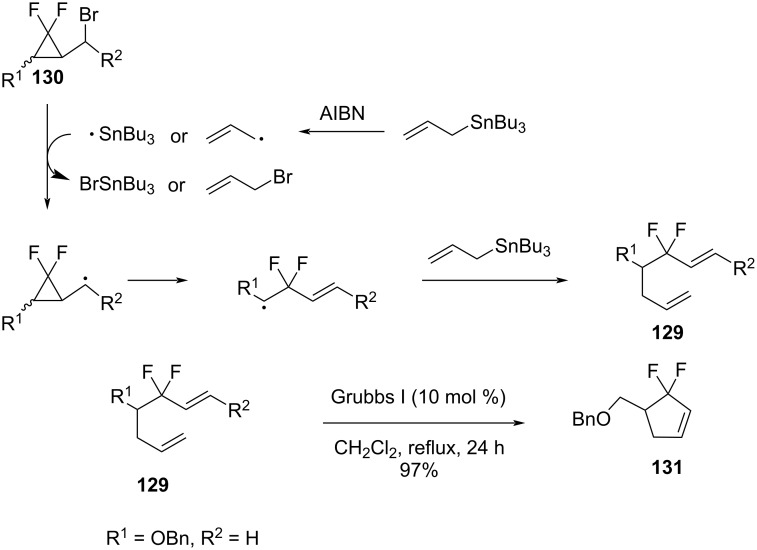
Ring opening of bromomethyl *gem-*difluorocyclopropanes **130** and formation of *gem*-difluoromethylene-containing cycloalkenes **131**.

Itoh et al. discovered the generation of 1,6-dienes **129** via the ring opening of bromomethyl-bearing *gem*-difluorocyclopropanes **130** due to the reaction with allyltributylstannane in the presence of AIBN (Scheme 56) [105]. The ring opening of the intermediate cyclopropylmethyl radical occurred with a cleavage of the distal C–C bond. The reaction proceeded regioselectively and in high yields. There was no difference observed between *cis* and *trans-*isomers in terms of the reactivity and yields. The resultant dienes **129** were used in ring-closing metathesis reactions to furnish *gem*-difluorocyclopentenes **131** in good to excellent yields [106].

**Scheme 57:**
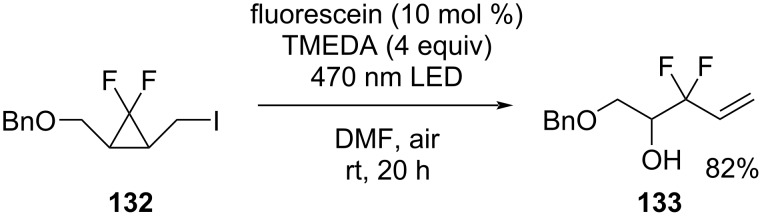
Ring-opening aerobic oxidation reaction of *gem-*difluorocyclopropanes **132**.

A convenient route to 2,2-difluoro-homoallylic alcohols **133** occurred by photo-irradiative aerobic oxidation (Scheme 57) [107]. The reaction proceeded by the light-mediated ring-opening reaction of *gem-*difluorocyclopropane **132** in the presence of an organic dye and the subsequent aerobic oxidation by an amine.

**Scheme 58:**
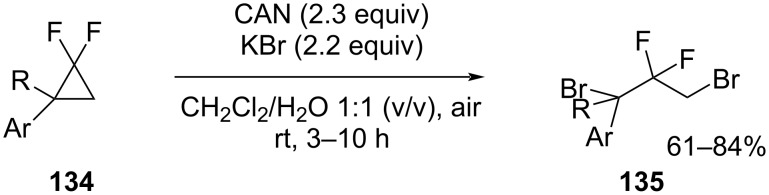
Dibrominative ring-opening functionalization of *gem*-difluorocyclopropanes **134**.

Single-electron oxidants such as cerium ammonium nitrate or K_2_S_2_O_8_ were used for the regiospecific ring opening of the simple *gem-*difluorocyclopropanes **134** (Scheme 58). The brominative ring-opening reactions of compounds **134** gave good yields of the dibromo derivatives **135** when KBr was employed in a DCM/H_2_O 1:1 (v/v) mixed solvent. Alternatively, the bromohydroxylation and bromoamidation were also achieved simply by changing the solvent system [108].

**Scheme 59:**
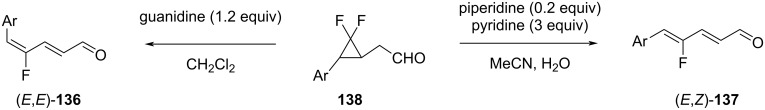
The selective formation of (*E*,*E*)- and (*E*,*Z*)-fluorodienals **136** and **137** from difluorocyclopropyl acetaldehydes **138**.

Stereodivergent sets of conditions were devised to produce stereodefined (*E*,*E*)- and (*E*,*Z*)-fluorodienals **136** and **137** in high yields by a base-induced cleavage of the weak distal bond of *gem-*difluorocyclopropyl acetaldehydes **138** (Scheme 59) [109].

**Scheme 60:**
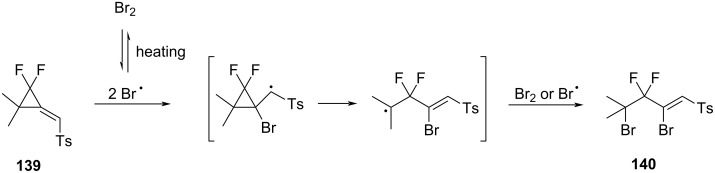
Proposed mechanism for the reaction of difluoro(methylene)cyclopropane **139** with Br_2_.

Xiao et al. studied the ring-opening reactions of difluoro(methylene)cyclopropane **139** with halogens and amines [110–111]. А number of fluorine-containing compounds were synthesized in this way. The reaction with bromine proceeded through the breaking of the distal bond of the cyclopropyl ring affording the final fluorine-containing compound **140** (Scheme 60) [111].

**Scheme 61:**
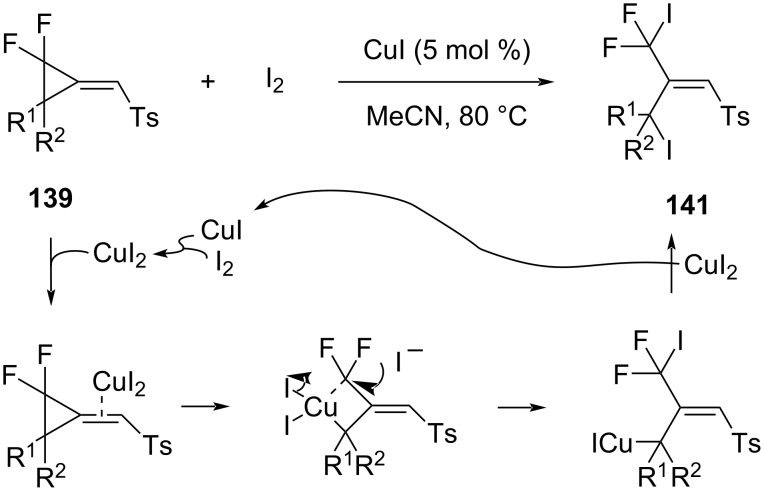
Thermal rearrangement of F_2_MCP **139** and iodine by CuI catalysis.

**Cleavage of the proximal bond:** Cheng investigated the ring-opening reactions of difluoro(methylene)cyclopropanes (F_2_MCPs) of type **139** (Scheme 61) [112]. The heating with iodine in the presence of CuI resulted in the cleavage of the proximal C2–C3 bond and the overall addition of a molecule of iodine to give products **141** in high yields.

**Scheme 62:**
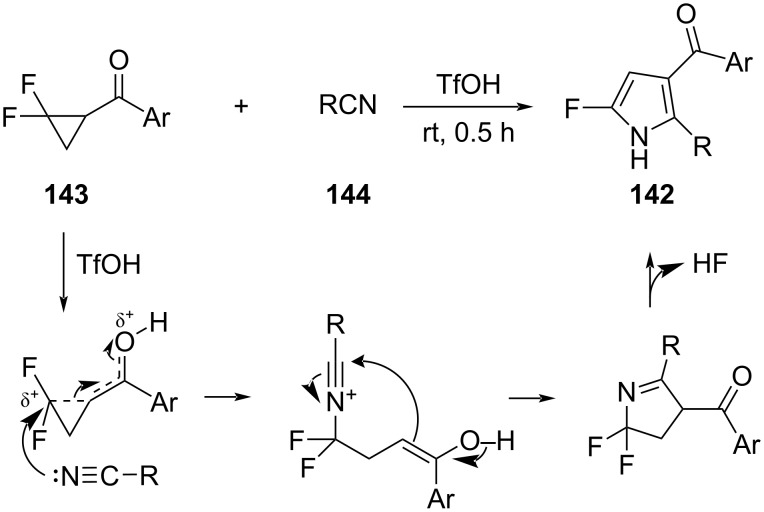
Synthesis of 2-fluoropyrroles **142**.

Xiao et al. described a direct synthesis of 2-fluoropyrroles **142** (Scheme 62) [113]. The reaction involved the *gem*-difluorocyclopropyl ketones **143** and nitriles **144**. It was proposed that the protonation of the ketone with triflic acid led to a partial ring opening of the *gem*-difluorocyclopropyl ketone to generate a carbocation-like center that was stabilized by the two attached fluorine atoms. The nucleophilic attack of the nitrile, followed by cyclization and aromatization could then give the pyrrole derivatives **142**.

**Scheme 63:**
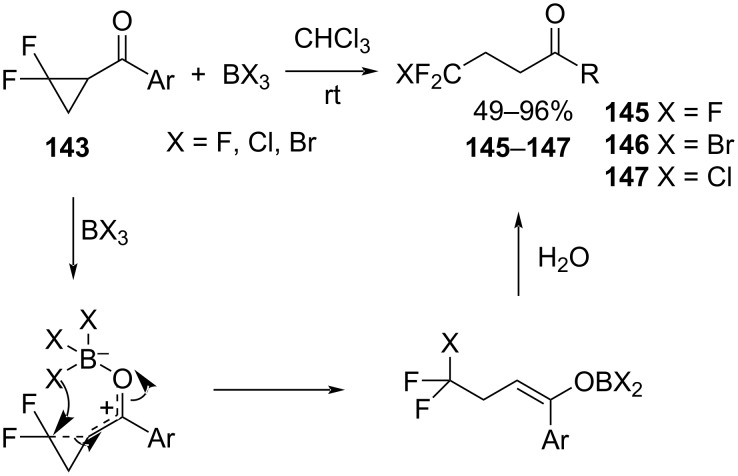
Ring opening of *gem*-difluorocyclopropyl ketones **143** mediated by BX_3_.

Later, Xiao et al. performed another ring-opening reaction of *gem-*difluorocyclopropyl ketones **143**, this time mediated by BX_3_ (X = F, Cl, Br, Scheme 63) [114]. In this transformation, BX_3_ played a dual role as both a Lewis acid catalyst and a source of the halide ion nucleophile. This reaction resulted in the generation of the trifluoromethyl ketones **145** and halodifluoromethyl ketones **146** and **147** in high yields. As in the previous reaction, a cleavage of the proximal bond accompanied the nucleophilic ring opening. The authors concluded that reactions mediated by weak acids resulted in the cleavage of the distal bond. This occurred by an S_N_2 attack at the less hindered carbon of the cyclopropyl group. In contrast to this, reactions mediated by strong acids led to the cleavage of the proximal bond by the generation of fluorine-stabilized carbocations (S_N_1 mechanism) [114].

**Scheme 64:**
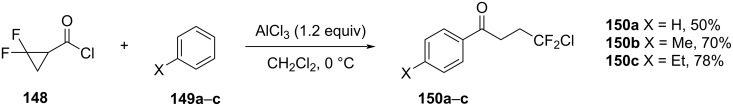
Lewis acid-promoted ring-opening reaction of 2,2-difluorocyclopropanecarbonyl chloride (**148**).

The Friedel–Crafts reaction of 2,2-difluorocyclopropanecarbonyl chloride (**148**) with arenes **149a–c** was accompanied by a proximal bond scission promoted by the strong Lewis acid AlCl_3_. This led to the formation of aryl 3-chloro-3,3-difluoropropyl ketones **150a**–**c** (Scheme 64) [115].

**Scheme 65:**
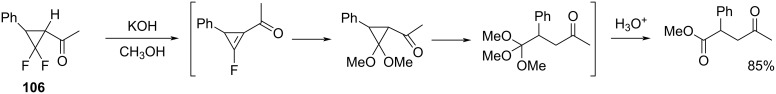
Ring-opening reaction of the *gem*-difluorocyclopropyl ketone **106** by methanolic KOH.

The gem-difluorocyclopropyl ketone **106** underwent a proximal bond cleavage in the reaction with methanolic KOH and methyl 4-oxo-2-phenylpentanoate was obtained in 85% yield after acid workup (Scheme 65) [97–98]. This contrasts with the previously discussed (Scheme 49) distal bond cleavage of ketone **106** in reactions with thiolate nucleophiles.

It is likely that an elimination of HF from **106** to form a monofluorocyclopropene intermediate took place under the more strongly basic conditions. This would facilitate the substitution of both fluorine atoms by methoxy groups prior to the ring opening, with the +M effect of the two MeO groups facilitating heterolysis of the proximal C–C bond.

**Transition metal-catalyzed ring-opening reactions:** Recently, the possibilities of using *gem-*difluorocyclopropanes in the synthesis of fluoroalkenyl-substituted compounds (monofluoroalkenes) have been actively studied. Great opportunities exist for the use of transition metal catalysis.

**Scheme 66:**
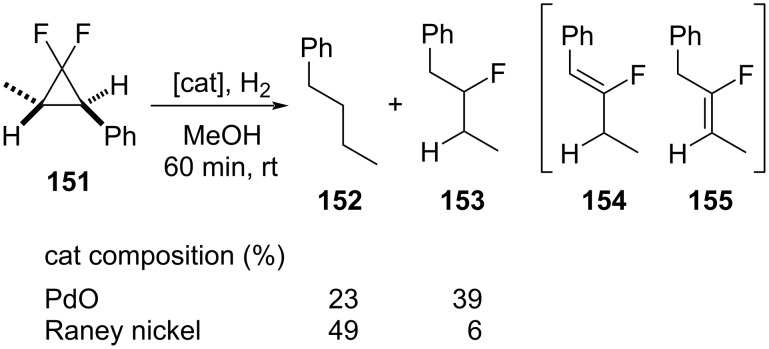
Hydrogenolysis of 1,1-difluoro-3-methyl-2-phenylcyclopropane (**151**).

The catalytic hydrogenolysis of 1,1-difluoro-3-methyl-2-phenylcyclopropane (**151**) led to the regioselective C2–C3 distal bond cleavage by the use of either palladium(II) oxide or Raney nickel as the catalyst (Scheme 66) [116]. Butylbenzene (**152**) and 2-fluoro-1-phenylbutane (**153**) were the main products, although the unsaturated intermediates **154** and **155** were also detected. The contribution of the fluorine substituents to the lengthening and weakening of the C2–C3 bond of the cyclopropane ring appeared to dictate the regioselectivity.

**Scheme 67:**
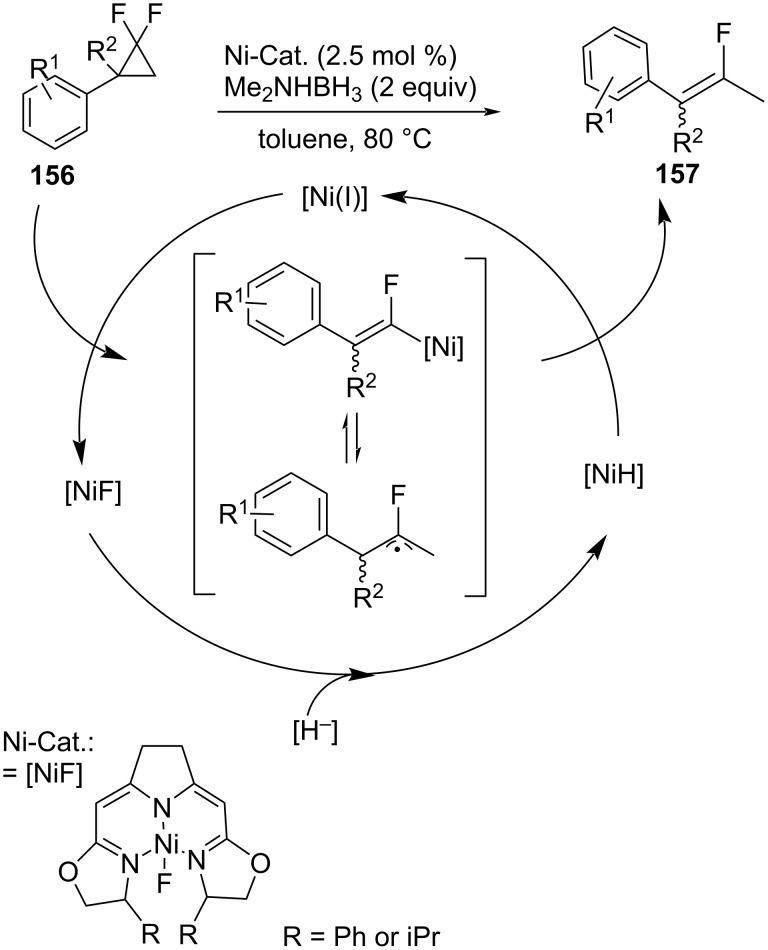
Synthesis of monofluoroalkenes **157**.

Monofluoroalkenes **157** were formed from the reductive ring opening of *gem-*difluorocyclopropanes **156** with dimethylamine·borane and catalyzed by nickel(II) fluorido complexes (Scheme 67) [117].

**Scheme 68:**
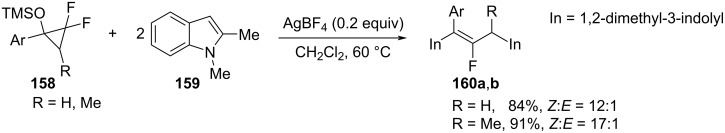
The stereoselective Ag-catalyzed defluorinative ring-opening diarylation of 1-trimethylsiloxy-2,2-difluorocyclopropanes **158**.

1-Trimethylsiloxy-2,2-difluorocyclopropanes **158** underwent a silver-promoted ring opening by the nucleophilic heteroaromatic 1,2-dimethylindole (**159**) to give aryl-substituted 2-fluoroalkenyl compounds **160a**,**b** (Scheme 68) [118]. An initial fluoride abstraction by Ag^+^ triggered the distal C–C bond cleavage to form an intermediate allylic cation which was the electrophile in a Friedel–Crafts reaction with **159**. The subsequent desilylation of the Friedel–Crafts product gave an α-fluorinated ketone intermediate which then reacted with a second equivalent of **159** in a (*Z*)-stereoselective, chelation-controlled process.

**Scheme 69:**
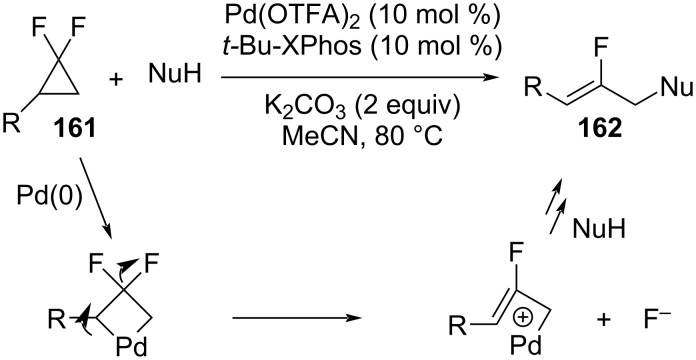
Synthesis of 2-fluorinated allylic compounds **162**.

Fu et al. presented a practical method to synthesize monofluorinated allylic scaffolds via a Pd-catalyzed C–C activation/C–F cleavage (Scheme 69) [119]. This ring opening of the *gem*-difluorocyclopropanes **161** occurred with both O- and N-nucleophiles. The resulting 2-fluorinated allylic products **162** were obtained in good yields and with high (*Z*)-selectivity. The proposed mechanism involved the oxidative addition of the distal C–C bond to palladium, followed by a nucleophilic attack at the less hindered carbon atom of a 2-fluorinated palladium–π-allyl complex.

**Scheme 70:**
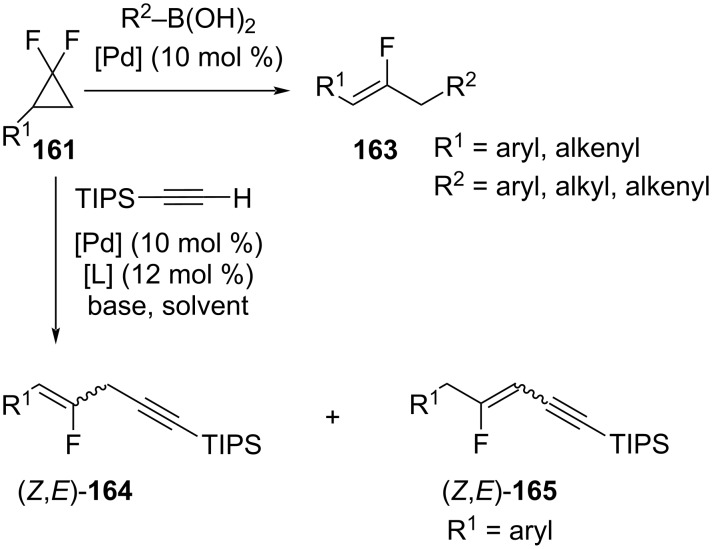
Pd-catalyzed cross-coupling reactions of *gem*-difluorinated cyclopropanes **161**.

Other examples of Pd-catalyzed ring-oрening reactions of *gem*-difluorocyclopropanes **161** are presented in Scheme 70. The first approach involved a Suzuki cross-coupling of the *gem*-fluorinated cyclopropanes **161** with boronic acids which afforded the monofluoroalkenes **163** [120]. Very recently, the groups by Gong and Fu [121] studied the Pd-catalyzed alkynylation of cyclopropanes **161** with terminal alkynes that led to the formation of the isomeric fluorinated enynes **164** and **165**.

**Scheme 71:**
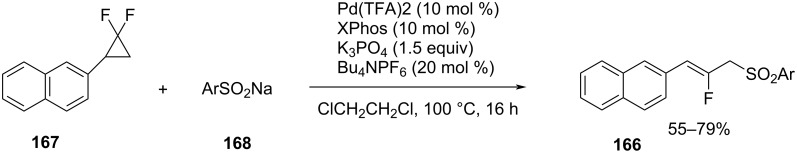
The (*Z*)-selective Pd-catalyzed ring-opening sulfonylation of 2-(2,2-difluorocyclopropyl)naphthalene (**167**).

Shortly before, a Pd-catalyzed ring-opening sulfonylation of *gem*-difluorocyclopropanes with the formation of 2-fluoroallylic sulfones **166** has been reported (Scheme 71) [122]. The reaction of 2-(2,2-difluorocyclopropyl)naphthalene (**167**) with sodium arylsulfinates **168** under palladium catalysis afforded the 2-fluoroallylic sulfones **166** in moderate to good yields with (*Z*)-selectivity. This method showed a good compatibility with a broad range of substrates and substituents.

As highlighted by these pioneering works [119–122], the direct Pd-catalyzed transformation of *gem*-difluorocyclopropanes to monofluoroalkenes is a promising approach towards the synthesis of fluorinated alkenes.

As is evident from the examples presented above, the ring opening of *gem*-difluorocyclopropanes occurs quite commonly under a variety of mild reaction conditions that are compatible with the presence of additional functionalities. The main reason for the ring opening is its inherent strain, with bond angles close to 60° instead of 109° that is normal for sp^3^ hybridized carbon atoms. The mechanisms of the ring opening that are favored in particular examples are determined by the additional substituents present on the cyclopropane ring, as well as the choice of reagents, catalysts, and conditions. The carbon atom 1 of the 1,1-difluorocyclopropane system, being attached directly to the fluorine atoms, has a significant partial positive charge and can be a site for nucleophilic attack. The neighboring carbon atoms also possess partial positive charges, albeit less pronounced. The combination of ring strain and the deficit of the electronic density leads to the possibility of ring opening by nucleophiles. As is the case for the nucleophilic opening of epoxides, the regiochemistry of this process is often controlled very effectively by the combined steric and electronic effects of the substituents attached to the ring, with a spectrum of S_N_1 and S_N_2-like reactivity possible.

Several different types of catalysts have proved effective in facilitating the ring opening of difluorocyclopropane derivatives. Lewis acids (e.g., group 13 halides and silver ions) can polarize carbonyl substituents and assist the loss of halide ions, leading to the formation of carbocation intermediates. Low-valent transition metals such as Pd(0) also have a valuable catalytic role, particularly because of their ability to participate in oxidative addition reactions and to form π-allyl complexes.

In the absence of nucleophiles, homolysis of the distal C–C bond takes place under the effect of high temperature. Such selectivity is caused by the possibility of the resonance stabilization of the biradical that is formed. If other reagents are absent, the biradical can rearrange and recombine, leading to isomerization of the starting material as was observed in the case of 1,1-difluoro-2,3-dialkylcyclopropanes. Further applications of free radical chemistry have developed through the use of radical initiators under comparatively mild conditions to form cyclopropylmethyl radicals, which can readily release their strain by opening to give homoallyl radicals.

*gem*-Difluorocyclopropanes, because of their ability to participate in such a diverse collection of ring-opening reactions and act as precursors for multifunctional products, both with and without fluorine, can play an exceptional role as intermediates for organic synthesis.

### 3 Biological activity of difluorocyclopropane derivatives

A further reason of interest in *gem*-difluorocyclopropanes stems from the unique influence of the fluorine substituents not only on the physicochemical properties, but also on the biological properties. Approximately one quarter of medicinal preparations, such as antibiotics, anticancer, and antimycotic preparations, contain at least one fluorine atom in their structure. The cyclopropane ring is a particularly attractive scaffold for incorporation in the design of pharmaceuticals because of its compactness, conformational rigidity, and the ability to support substituents in well-defined regions of three-dimensional space.

**Figure 1:**
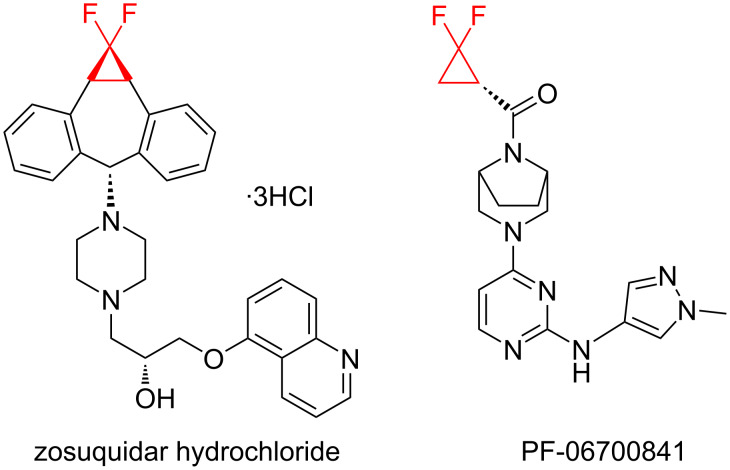
Structures of zosuquidar hydrochloride and PF-06700841.

There is a high risk of failure of cancer chemotherapy due to the development of multidrug resistance which can arise by an overexpression of P-glycoprotein, an energy-dependent drug efflux pump. In this case increased dosages of therapeutics are required, leading to dangerous toxicity and high risk of death. Therefore, modulators have been developed in order to restore the sensitivity to chemotherapy. One such modulator is the *gem-*difluorocyclopropane derivative LY335979·3HCl (zosuquidar hydrochloride, Figure 1) [123].

PF-06700841 (Figure 1) is a dual protein kinase inhibitor that targets cytokine signaling pathways associated with autoimmune disorders such as plaque psoriasis [124–125]. The non-covalent binding between this inhibitor and the target proteins has been characterized by single crystal X-ray diffraction; the difluoromethylene unit was found to project into the phosphate-binding loops of the kinases’ ATP binding sites.

Another use of *gem*-difluorocyclopropanes has been in the preparation of nucleoside analogs, which can act as chemotherapeutic agents. Carbocyclic nucleosides can have antiviral activity. Therefore, Csuk and Eversmann [32] studied the synthesis of *gem*-difluorocyclopropyl carbocyclic nucleosides for use against HIV.

**Scheme 72:**
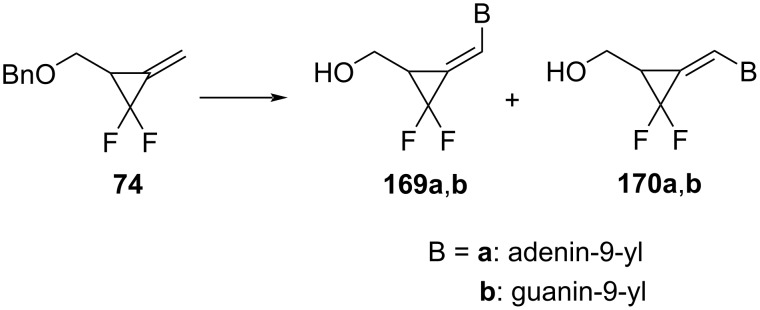
Synthesis of methylene-*gem*-difluorocyclopropane analogs of nucleosides.

Furthermore, Wang et al. reported the biological activity of the methylene-*gem*-difluorocyclopropane analogs of nucleosides **169a**, **169b**, **170a**, and **170b** that were obtained from methylene-*gem-*difluorocyclopropane **74** (Scheme 72) [76]. Compound **169a** was active against human cytomegalovirus (HCMV) in human foreskin fibroblast cells. Both compounds **169a** and **170a** had antitumor activity, but derivative **169a** was found to be more selective in comparison to its isomer **170a**.

**Figure 2:**
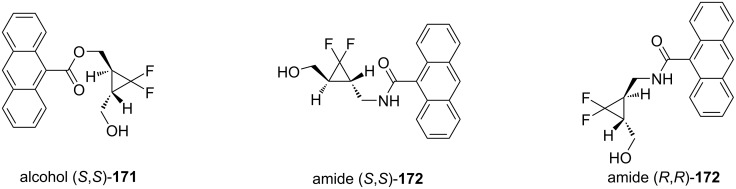
Anthracene-difluorocyclopropane hybrid derivatives.

Recently, there has been much interest in the synthesis of organic compounds that can cleave DNA following photoirradiation. Ninomiya et al. described anthracene–difluorocyclopropane hybrids, which were modified in order to maximize DNA cleavage [126]. DNA damage was induced due to the radical decomposition of the cyclopropane ring. The active derivatives included compounds (*S*,*S*)-**171**, (*S*,*S*)-**172**, and (*R*,*R*)-**172** (Figure 2).

**Figure 3:**
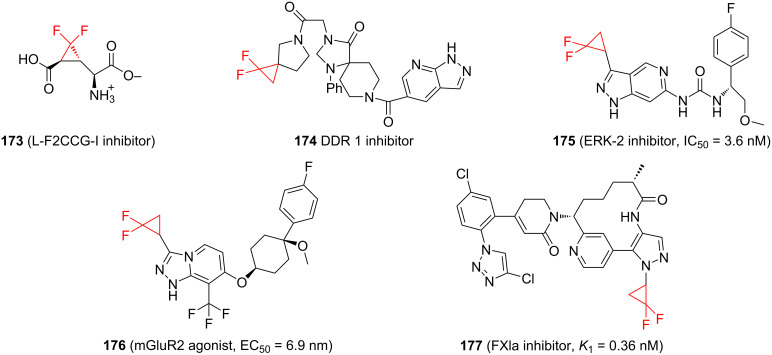
Further examples of difluorcyclopropanes in modern drug discovery.

Further examples highlighting the importance of *gem-*difluorocyclopropanes in modern drug discovery are shown in Figure 3. Compound **173** was selected as a selective agonist for the metabotropic glutamate receptor 2 with antiepileptogenic effects [127]. Compound **174** is a discoidin domain receptor 1 inhibitor with the potential of being applied for the treatment of cancer and inflammation related disorders [128]. Compound **175** is an extracellular signal-regulated kinase 2 inhibitor with potential as an anticancer drug [129]. Compound **176** has potential for the treatment of neurological and psychiatric disorders [130] and compound **177** is an FXIa inhibitor with anticoagulant activity [131].

Another reason for the great interest in difluorocyclopropane derivatives arises also from the compounds’ ability to control the plant growth and fruit ripening. Ethylene is a plant hormone, which controls fruit ripening [132], seed germination, and leaf senescence. It is biosynthesized from 1-aminocyclopropane-1-carboxylic acid (ACC) and under stress conditions, ethylene can be produced in excess amounts, leading to senescence, chlorosis, and abscission. The ACC analog 1-amino-2,2-difluorocyclopropane-1-carboxylic acid can inhibit the enzyme ACC deaminase, which stops fruits from ripening, prevents the loss of leaves, etc. With the help of this substance, the shelf life of vegetables and fruits can be increased.

In addition, *gem*-difluorocyclopropanes can be used in agriculture against spider mite (*Tetrahychus urticae*), diamondback moth (*Plutella xylostella*), worm (*Spodoptera littoralis*) and Mexican caryopsis (*Epilachna varivestis*) [133].

## Conclusion

*gem*-Difluorocyclopropanes were discovered to be effective substrates for the generation of medicinal and bioactive materials. Lately, more studies have been made regarding the inclusion of this motif into drug structures. The presence of the geminal fluorine atoms was associated with the increase of the lipophilicity, bioavailability, metabolic stability, and the binding affinity of biologically active materials. For instance, gem-difluorocyclopropanes have been incorporated into nucleoside analogs, which act as antiviral agents and where one of the roles of the fluorine is to act as a hydrogen-bond acceptor. Antimicrobial agents, amino acids, and drugs used for the control of chemotherapy sensitivity also include difluorocyclopropane derivatives.

Therefore, the synthesis of difluorocyclopropanes on a large scale and in safe conditions is a subject of great importance and relevance. This review considered numerous preparation methods for *gem-*difluorocyclopropanes. However, the most popular approach is based on the use of the reactive intermediate difluorocarbene, which can participate in stereospecific [2 + 1]-cycloadditions with alkenes. Difluorocarbene addition reactions have been complemented by other synthetic methods, some of which have provided optically active difluorocyclopropanes, e.g., by functional group interconversions involving enzyme-catalyzed esterifications and catalytic asymmetric hydrogenation reactions of difluorocylopropenes.

Thermal rearrangements and ring-opening reactions place difluorocyclopropanes at the crossroads in the synthesis of useful compounds. These processes often lead to the cleavage of the distal C–C bond and the cleavage of the proximal bond has also been observed but the electronic and steric factors determining the regioselectivity are now well appreciated.

Recent strategies including the transition metal-catalyzed transformations of highly substituted difluorocyclopropanes opened the door for the development of asymmetric approaches with the use of chiral ligands and chiral reagents. This is of great importance for the discovery and the development of new bioactive compounds. There have been made many discoveries regarding the synthesis and reactivity of difluorocyclopropanes in the last 60 years. However, more work is needed to develop catalytic enantioselective processes, both, for the cyclopropane ring formation and ring opening. These compounds have yet to receive the attention that has provided effective methods for the catalytic asymmetric transformations involving other three-membered rings, such as epoxidation, Simmons–Smith cyclopropanation, and epoxide opening. The possibility of using complexes of abundant transition metals such as copper as reagents for difluorocarbene transfer [90] raises our hopes for future developments in this area.
